# Psychometric properties and measurement invariance of the Perth Alexithymia Questionnaire Short Form (PAQ-S) in Arabic, English, Italian, Spanish, Turkish, and Ukrainian

**DOI:** 10.3934/publichealth.2025042

**Published:** 2025-08-13

**Authors:** Olga Malas, Giulia Colombini, Anastasiia Shyroka, Dayo Omotoso, Asiye-Şengül Avşar, Nada Mallah Boustani, Mirko Duradoni, Angel Blanch

**Affiliations:** 1 Department of Psychology, Sociology and Social Work, University of Lleida, Avinguda de l'Estudi General, 4, 25001 Lleida, Spain; 2 Department of Education, Languages, Interculture, Literatures and Psychology, University of Florence, Via di San Salvi, 12, Building 26, 50135 Florence, Italy; 3 Department of Psychology and Psychotherapy, Ukrainian Catholic University, Sventsitskogo 17, 79011 Lviv, Ukraine; 4 Department of Human Anatomy, Redeemer's University, Ede, Osun State, Nigeria; 5 Department of Measurement and Evaluation in Education, Recep Tayyip Erdoğan University, Campus Zihni Derin - Fener Mahallesi 53100 Rize, Türkiye; 6 Faculty of business and management, Saint Joseph University, PO BOX 17-5208 Mar Mikhael, 1104 2020 Beirut, Lebanon

**Keywords:** alexithymia, type D personality, PAQ-S, classical test theory, network analysis, psychometrics

## Abstract

Alexithymia is a personality trait with significant clinical impact worldwide. It is a relevant transdiagnostic risk factor for a wide range of psychopathologies, including depression, anxiety, eating and substance use disorders, and other psychosomatic conditions. This underscores the importance of having validated instruments to measure alexithymia, particularly brief scales suitable for quick and practical applications. The Perth Alexithymia Questionnaire (PAQ-S) has shown promising results in this context. However, few cross-cultural studies have validated its use, which is the aim of the present study. To this end, a sample of 2535 university students was recruited (*mean* age = 20.59 years; *SD* = 2.04; 26.75% males and 73.25% females) from Spain (*n* = 388), Italy (*n* = 376), Lebanon (*n* = 487), Nigeria (*n* = 561), Türkiye (*n* = 410), and Ukraine (*n* = 313). The PAQ-S and the Type D Personality Scale (DS-14) – designed to assess Negative Affectivity (NA) and Social Inhibition (SI) – were administered. The Classical Test Theory (CTT) and Network Analysis (NwA) were applied. The confirmatory factor analysis yielded satisfactory results in all cases, with an adequate internal consistency. Metric invariance was obtained between genders and cultures. Additionally, several of the analysed countries presented strict invariance. Therefore, the data obtained in these countries can be compared and their results extrapolated between them. The NwA supports the data obtained through the CTT, as well as the independence of alexithymia, NA, and SI constructs. Hence, the scale proved to be useful for its intended aim and may be useful to monitor alexithymia in large-scale health campaigns.

## 1. Introduction

Alexithymia can be described a difficulty in identifying one's own feelings (DIF), difficulty in describing feelings (DDF), and an externally oriented thinking style (EOT) marked by a tendency to prioritize external information processing over introspective emotional awareness [1]. It is characterized by deficits in affect regulation, such as a difficulty in identifying, expressing, and managing emotions, as well as a limited access to adaptive resources such as fantasy, social support, and a tolerance of emotional distress [2]. These limitations may play a role in the onset or persistence of both physical and psychological illnesses, manifesting in somatic symptoms, compulsive, or addictive behaviors, and the dysregulation of physiological systems such as the immune, endocrine, and autonomic systems [2],[3]. Initially identified in patients with various psychosomatic conditions, alexithymia has subsequently been recognized across a broad spectrum of medical and psychological disorders, including rheumatoid arthritis, hypertension, inflammatory bowel disease, irritable bowel syndrome, peptic ulcers, cancer, diabetes, mood disorders, personality disorders, depression, anxiety, eating or substance use disorders, and other psychosomatic disorders [2]–[8].

From a public health perspective, individuals with pronounced alexithymic traits represent a vulnerable group, as their limited ability to identify and recognize the severity of symptoms often delays the seeking of medical attention, which negatively affects treatment times and may, as an example, reduce survival rates in cases of acute myocardial infarction [4],[9].

Hence, the interest in its study has increased in recent years and has been made possible by the development of psychometric measurement tools. The main measurement tools are the 20-item Toronto Alexithymia Scale (TAS-20; [10]), the Bermond-Vorst Alexithymia Questionnaire (BVAQ; [11]), and the Perth Alexithymia Questionnaire (PAQ; [12]). These measures have the advantage of allowing the construct to be measured at the facet level; however, their lengths are a disadvantage, thus limiting their use in clinical contexts, longitudinal applications, or complex research settings in which alexithymia represents just one among several variables under investigation [13]. To address this issue, an abbreviated form of the PAQ (as PAQ-S) has been developed, with only 6 items covering the DIF, DDF, and EOT facets of the construct. It was developed to yield an overall score based on all items, thus serving as a general indicator of alexithymia [13]. Its development was based on evidence that much of the variance in the items of existing alexithymia measures was due to a strong general factor [14].

### 1.1. Alexithymia and type D personality

Patients with alexithymia tend to exhibit high levels of Type D personality, which has called the need to measure both constructs into question [15]. According to Denollet et al. [16], Type D personality refers to a distress-related personality pattern defined by the co-occurrence and interactive effect of two key traits: negative affectivity (NA) and social inhibition (SI), where the latter involves difficulties in expressing thoughts and emotions. Therefore, it has been postulated that they could be different measures of the same construct (high neuroticism and low extraversion), with alexithymia potentially explaining the structure of Type D personality as a stable tendency to cope with emotions, which is fundamentally based on affective dysregulation [15]. This hypothesis was supported by data that indicated high correlations between the two constructs [17], both being associated with the components of the five-factor model, particularly neuroticism and extraversion (e.g., [18],[19]), and being risk factors for depression, anxiety [20],[21], and a range of physical pathologies [20],[22]. Although, studies have applied an exploratory factor analysis (EFA) and structural equation modelling (SEM) to Scottish students [23], Iranian students [24], and Italian outpatients [25] to demonstrate the independence of the constructs, additional studies in larger populations would be necessary.

### 1.2. Alexithymia and cultural differences

The viability of measuring alexithymia in cultures, such as those in Asia, has also been questioned [26]. The significance of alexithymia as a predictor of psychopathological disorders lies in the idea that the ability to express emotions is crucial for psychological health [27]. However, in Asian cultures, the interpersonal context prevails over private feelings and their expression [28], and their population tends to self-report higher levels of alexithymia compared to individuals in Western countries (e.g., [26],[29],[30]). This could be due to marked differences in the level of alexithymia in the sample, but also due to cultural differences or issues with the cross-cultural adaptation of measurement tools [28]. A similar situation might apply to certain African cultures, such as the Nigerian culture, where self-control, emotional moderation, and an adherence to social norms are also highly valued [31]. Thus, culture can influence the responses to scales and questionnaires intended to measure alexithymia, thus suggesting that further studies in other populations are essential.

### 1.3. Psychometric analysis of the PAQ-S

Psychometric analyses of the PAQ-S have been conducted in its original English version with samples from the general populations of American and Australian university students [13], in the Polish language with a general population sample in Poland [32], and in the Russian language with a general population sample in Russia [33]. Both adaptations confirmed the results obtained with the original PAQ-S, thereby supporting its one-factor structure, internal consistency, convergent and divergent validity, and invariance across genders and age groups [32],[33]. Additionally, the PAQ-S showed a good predictive capacity for psychopathological symptoms and well-being [13],[32],[33].

In the first study, with a general population sample from the United States, an EFA was conducted to elucidate its structure using principal axis factoring, direct oblimin rotation, and optimal factor number extraction based on a parallel analysis; this was followed by a confirmatory factor analysis (CFA) in a sample of Australian university students [13]. Among the Polish and Russian general population samples, a confirmatory factor analysis with maximum likelihood estimation, robust standard errors, and the Satorra-Bentler scaled chi-square test was used [32],[33]. Both studies allowed for error terms across three pairs of items (the 1 and 2, the 3 and 6, and the 4 and 5), arguing that these reflected the fundamental theoretical framework of the scale, thereby connecting the two items related to the evaluation of negative emotions, the two items concerning positive emotions, and the two specific EOT items.

### 1.4. Psychometric Network Analysis (NwA)

The psychometric studies previously performed for the PAQ-S have focused on traditional latent variable approaches to explore the factor structure and their psychometric properties by applying the classical test theory (CTT). However, traditional latent variable approaches have been criticized due to inherent flaws based on the premise of local independence among symptoms, which may not be realistic, and because the latent structure may not be categorical [34],[35]. Consequently, in recent years, network psychometrics has gained prominence as a complement to traditional latent variable methods, with growing applications in the areas of psychopathology and psychometrics [36],[37]. Moreover, this analytical approach offers an in-depth graphical depiction of the intricate relationships between variables and a deeper understanding of their interaction, which is difficult to achieve with the traditional techniques. A NwA allows for the visualization of direct and indirect associations between variables, the identification of key nodes within a network framework, and bridges nodes or connectors between variables. In turn, the variables, which are represented as nodes, are organized into ranked and interconnected subnetworks, creating clusters akin to the latent components of the variables [38], thus providing information on the extent to which they represent a dimension and to show whether the components effectively assess a construct [39]. Thus, a psychometric NwA could offer fresh insights to assess the structure and dynamics of measurements in mental health contexts, including personality traits such as alexithymia.

### 1.5. The current study

The present study aims to evaluate the psychometric properties of the PAQ-S in a cross-cultural sample from six populations with different cultures and languages (Arabic, English, Italian, Spanish, Turkish, and Ukrainian). The psychometric properties will be examined by the CTT and NwA approaches, thus allowing for a comprehensive evaluation of its reliability, factorial structure, and measurement invariance across these diverse cultural groups.

Drawing on the research cited in the background section, the present study formulated the following hypotheses:

(1) the expected one-factor structure of the PAQ-S fits the data well;

(2) the construct of alexithymia measured with the PAQ-S shows independence from the Type D personality construct measured with the Type D Personality Scale (DS14), that separately assesses trait negativity and social inhibition;

(3) the PAQ-S has a good internal consistency across all analysed samples;

(4) higher alexithymia scores on the PAQ-S positively correlates with markers of Type D personality; and

(5) the questionnaire demonstrates measurement invariance between genders but not between the different cultures analysed.

## 2. Materials and methods

### 2.1. Participants

The participants were university students recruited from Lebanon (*n* = 487; *Mean* age = 20.25, *SD* = 1.13; 58.7% women), Nigeria (*n* = 561; *Mean* age = 19.95, *SD* = 2.36; 68.8 women), Italy (*n* = 376; *Mean* age = 21.78, *SD* = 1.79; 79.0% women), Spain (*n* = 388; *Mean* age = 21.37, *SD* = 2.07; 79.9% women), Türkiye (*n* = 410; *Mean* age = 21.53, *SD* = 1.41; 79.8% women), and Ukraine (*n* = 313; *Mean* age = 18.69, *SD* = 1.34; 80.2% women). The final sample (*N* = 2535) included only students aged 18 to 25 years (*M* = 20.60, *SD* = 2.04), with 73.3% identifying as female and 26.7% identifying as male.

The sample size in each country was determined based on the most stringent criteria among the recommended guidelines for the planned analyses. Specifically, we adopted the minimum recommended by Tabachnick and Fidell [40], which suggests at least 300 participants to ensure stable and reliable results in the factor analysis within the framework of the CTT. Additionally, this threshold satisfies the criterion proposed by Nunnally and Bernstein [41] (a subject-to-variable ratio of at least 10:1) given the number of items in our instrument. Moreover, this sample size aligns with recommendations for the NwA, as simulation studies previously indicated that samples of approximately 250 participants are adequate to estimate networks with up to 25 nodes using continuous data [42]. Therefore, we ensured that each country's sample met or exceeded these thresholds.

### 2.2. Procedure and ethics approval of research

To ensure cross-national comparability in terms of educational background, cognitive capacity, and age, university students were recruited as participants in each country. Data collection was performed through online surveys, with all instruments administered in the respective official languages of the participating nations: Arabic, English, Italian, Spanish, Turkish, and Ukrainian. Since the PAQ-S is a nested scale of the PAQ and has already been validated in the languages of the study, there was no need to proceed with cross-cultural adaptation.

The participants in the study were enrolled through a digital data collection process. A web-based link to the study questionnaire was distributed via email to all identified potential participants. The study was disseminated by sending emails to professors at various universities across the participating countries. The selection of professors was random and included representatives from different academic disciplines, such as the sciences, humanities, and technical fields, with the aim of promoting a diverse and heterogeneous sample of university students. The message informed them of the study's objectives and included a link to the questionnaire and the informed consent document. The professors who agreed to collaborate voluntarily forwarded the invitation to their students. Student participation was also voluntary. Consequently, it was not possible to ensure a uniform percentage of students from each field of study in every country. For this reason, and considering the scope and main objectives of the present study, the departments or faculties involved, as well as the distribution of students by academic area, were not reported.

Before completing the questionnaire, all potential participants received detailed information about the purpose of the research and the confidentiality measures applied to the data collected. Participation was entirely voluntary and without any financial compensation. Informed consent was obtained from each individual prior to their inclusion in the study. Ethical approval for the research was obtained from the Ethics Review Committee of the University of Lleida – Spain (Ref.: CERT2023).

### 2.3. Instruments

Sociodemographic variables (age and sex) were recorded. Subsequently, all participants completed the PAQ-S [13] and the DS14 [43] questionnaires.

The PAQ-S is a unidimensional scale consisting of 6 items. The PAQ-S items can be seen in Table S1 within the supplementary. It is scored on a 7-point Likert scale, ranging from 1 (completely disagree) to 7 (completely agree). Higher scores indicate higher levels of alexithymia. In the total sample of this study, the Cronbach's α obtained was 0.83, which indicated good internal consistency. In this study, the scale from Italy, Lebanon, Nigeria, Spain, Türkiye, and Ukraine yielded Cronbach's α coefficients ranging between 0.79 and 0.87.

The DS14 is a self-report instrument composed of 14 items, specifically developed to assess the Type D (distressed) personality construct. It is structured around two distinct but interrelated dimensions: NA and SI. The NA subscale encompasses items 2, 4, 5, 7, 9, 12, and 13, which capture a tendency to experience negative emotions such as dysphoria, worry, and irritability. The SI subscale includes items 1, 3, 6, 8, 10, 11, and 14, and evaluates discomfort in social interactions and avoidance of social engagement. Each item is rated using a 5-point Likert scale, ranging from 0 (not true at all) to 4 (completely true), thus reflecting the degree to which each statement applies to the respondent. Notably, items 1 and 3 are reverse-coded to control for response bias. In this study, the internal consistency measured by Cronbach's α was 0.86 for the NA subscale and 0.80 for the SI subscale in the total sample. Across the individual country samples, the Cronbach's α values ranged from 0.83 to 0.90 for the total scale, 0.80 to 0.89 for NA, and 0.66 to 0.88 for SI.

### 2.4. Statistical analysis

Using SPSS, v.28, the descriptive and frequency statistics were applied to the scales and sub-scales. Additionally, the skewness, kurtosis, and Kolmogorov-Smirnov test values were examined to evaluate the data's adherence to a normal distribution. Then, a one-way analysis of variance (ANOVA) was employed to examine differences in the scale scores between groups defined by country and gender in order to identify whether these demographic variables influenced the measured outcomes.

The CTT was carried out using the JASP, v.0.18, package. For the CFA, the Diagonal Weighted Least Squares (DWLS) and Weighted Least Squares Mean and Variance Adjusted (WLSMV) estimation methods were applied.

In the studies conducted by Larionow et al. [32],[33], a CFA was performed using the maximum likelihood estimation (MLE) with robust standard errors, along with the Satorra–Bentler scaled test statistic (MLM). The MLE has limitations when analyzing data that do not follow a continuous normal distribution [34]. Larionow et al. [32],[33] proposed that all the analysed variables were reasonably normally distributed. Preece et al. [13] did not specify the methodology used but assumed the normality assumption. The use of robust MLE could be considered a viable alternative in this case, but it is not always recommended for the analysis of categorical data [44]. In this study, only the samples from Nigeria and Ukraine met this assumption, whereas neither the total sample nor the rest of the individual samples did. This may explain why, in this study, some fit indices (such as RMSEA) are inadequate when applying the MLE or MLM. In these cases, the DWLS method was a better alternative [44]–[46]. Recent studies also recommended the use of the WLSMV method to handle the ordinal data [47]. Unlike the DWLS method, the WLSMV method adjusts both the mean and variance, thus providing more robust standard errors and model fit indices.

Consequently, a CFA with DWLS and WLSMW estimation methods was performed on both the total sample and each country sample to evaluate the adequacy of the one-factor structure. To assess the model fit, the following indices were considered: *X^2^*/gl, Comparative Fit Index (CFI), Tucker-Lewis's index (TLI), Root Mean Square Error of Approximation (RMSEA), and Standardized Mean Square Residual (SRMR). The acceptance criteria for an acceptable fit were as follows: *X^2^*//gl < 5.00; TLI and CFI > 0.90; RMSEAs < 0.08; and SRMR ≤ 0.08 [48].

To assess the internal consistency and reliability of the scale, both Cronbach's α and McDonald's ω were calculated, with α-values > 0.80 and ω-values > 0.75 being preferable [49]. To assess the concurrent validity, a correlation analysis was conducted between the PAQ-S scores and the NA and SI scores obtained using the DS14, with *r* ≥ 0.300 as moderate result and *r* ≥ 0.500 as good result [50].

The measurement invariance across the participating countries and sexes (configural, metric, scalar, and strict) was assessed using multi-group confirmatory factor analysis (MGCFA). The measurement invariance was analyzed between pairs of samples. Although a simultaneous analysis of all groups can allow the assessment of global model invariance, this approach was chosen because it does not reveal between which specific groups invariance fails. A pairwise analysis enables the precise identification of which country pairs maintain invariance and which do not. This facilitates culturally or regionally specific comparisons (e.g., between Mediterranean countries, European countries, or countries from different continents) and allows for the construction of partial equivalence maps across culturally diverse samples. To examine the factorial invariance between these groups, we compared the equality of key model parameters, including the structural configuration, factor loadings, intercepts, and error terms, within the measurement model. This analysis helps determine whether the factor structure remains consistent across different countries and sexes, thus ensuring that the scale functions equivalently in these diverse groups [51]. Building on the established model, the invariance constraints were applied in a stepwise manner. For the configural invariance, the factors and structural configuration were held constant across all groups. To calculate the metric, scalar, and strict invariance, one must progressively equalize the factor loadings, item intersections, and measurement errors. Models with invariance constraints were compared to the unrestricted model using fit indices, with the criteria for invariance being ΔCFI ≤ 0.01 and ΔRMSEA ≤ 0.015 [52].

Furthermore, a NwA was conducted using the JASP, v.0.18, package. First, to elucidate the independence between constructs, an undirected network model was estimated by loading the items of PAQ-S and DS14 as nodes in the network. A Mixed Graphical Model (MGM) was used with a regularized estimation technique that applies the Extended Bayesian Information Criterion (EBIC) to select the tuning parameter [39]. An MGM was chosen because it is suitable to handle data from both continuous and categorical variables [53]. The EBIC estimation method was employed to estimate the model parameters and to identify the most suitable network structure because it helps to prevent overfitting by choosing simpler and more parsimonious models [39]. In an MGM, variables were depicted as nodes within a graph, while the relationships or connections between these variables were represented as edges using the Pairwise Markov Random Field (PMRF) approach. These edges indicated the presence and strength of associations between variables. For a node's strength to be significant, it must exceed the recommended threshold of 0.5 [39]. The edges were undirected and define a conditional dependency given all other nodes in the network [54]. The JASP package adopted the Fruchterman-Reingold algorithm to organize the network according to the strength of connections between nodes [55]. Therefore, the variables, depicted as nodes, were organized into ordered and interconnected subnetworks, thus creating clusters that resemble the latent components of the variables [41]. This arrangement offers insights into how well the variables align with a particular dimension and helps assess whether the components effectively capture the construct being measured [42].

Subsequently, the network structure of the PAQ-S was examined in accordance with the guidelines provided by Epskamp et al. [38], thereby following a three-step process. In the first step, the network model was estimated. The second step involved analyzing the network structure by evaluating its centrality indices. In the third step, the stability of the network parameters was evaluated. Thus, following the aforementioned methodology, the most appropriate network structure was generated and selected, and the network was organized according to the strength of connections between nodes, where fundamental and central nodes are those that exhibit strong impacts on the overall network due to a high number of edges [55]. In the second step, four centrality indices (betweenness, closeness, strength, and expected influence) were calculated to assess the significance of each node. The strength index represents the sum of the absolute weights of a node's direct connections, with higher values indicating stronger or more numerous associations with other nodes. Betweenness reflects how often a node lies on the shortest paths between pairs of other nodes, meaning that higher values indicate a bridging or intermediary role within the network. Closeness is defined as the inverse of the average distance from a node to all others, which allows for the identification of nodes that are, on average, closer to the rest, and therefore more accessible. Finally, the expected influence is like strength but takes the sign of the weights (positive or negative) into account, thus providing an estimate of the net effect a node may exert on the overall system. As Epskamp et al. [38] and others, to facilitate interpretation of the results, the centrality indices are presented as standardized *z*-scores in this study. *Z*-scores represent the number of standard deviations a given value is above or below the mean, and they can vary across a wide range, although most values typically fall approximately between −3 and +3. Values close to or below zero indicate low or negligible centrality, while higher positive values reflect greater relative importance of the nodes within the network. There is no absolute threshold to define a *z*-score as “high” or “good”, but scores above +1 are generally interpreted as indicating high centrality, and those between +0.5 and +1 as indicating moderate centrality. Comparing the scores within the same network is essential to identify the most central and relevant nodes. Nodes with higher centrality values were identified as the most influential within the network [39]. Finally, following the Hevey [54] recommendations, the stability of both centrality indices and edge weights were assessed through the subset bootstrapping method, which involved performing 1000 iterations to ensure reliable estimates. A correlation stability coefficient of ≥ 0.7 was considered an appropriate threshold for the centrality indices [39].

## 3. Results

### 3.1. Descriptive statistics

The mean and standard deviation results for each PAQ-S item across the different subgroups (sex and countries) can be found in Table S2 within the supplementary.

Table 1 presents the results for the descriptive statistics, correlations, reliability coefficients, and fit indices for the PAQ-S total scale, broken down by sex and country. The skewness and kurtosis values were within the range of −1 and +1, which suggests a normal distribution. However, based on the results of the K-S test, it can be concluded that only the sample from Nigeria and Ukraine presented a normal distribution. The total sample and other samples did not follow a normal distribution. The K-S test tends to be highly sensitive and may detect minor deviations when large samples are analyzed. In such cases, the skewness and kurtosis values may be considered more reliable. However, the samples used were not excessively large. Furthermore, although Likert-type scales are numerical, they are ordinal in nature and do not guarantee equal intervals between values. Therefore, it cannot be assumed that they represent interval-level data, which is required by traditional parametric methods. For this reason, and based on the results, it was considered more appropriate to adopt a conservative approach using non-parametric analyses. Consequently, Spearman's rho correlations were estimated between the levels of the PAQ-S and the levels of NA and SI obtained with the DS14.

In the ANOVA analysis, significant differences (*p* < 0.001) were obtained between countries but not between sexes.

The samples from Nigeria and Lebanon showed the highest mean scores and the samples from Italy and Turkey exhibited the lowest mean scores. Spain, Ukraine, and Turkey occupied intermediate positions, which suggests clear and consistent variations in the responses according to cultural, social, or economic factors specific to each country.

Table S2 presents the mean and standard deviation for each of the PAQ-S items across the countries studied. As can be observed, Lebanon and Nigeria consistently showed the highest means across all items. Item 1 (When I'm feeling bad (feeling an unpleasant emotion), I can't find the right words to describe those feelings) scored the highest in all countries, thus indicating that this is the greatest perceived emotional difficulty. Item 3 (I tend to ignore how I feel) was generally the second highest scoring item, except in Italy and Turkey, where item 4 (When I'm feeling good (feeling a pleasant emotion), I can't find the right words to describe those feelings) took second place. Overall, the differences suggest that while the difficulty expressing negative emotions is constant, the attention to and differentiation of positive emotions vary culturally.

**Table 1.** Descriptive statistics, Correlations, Reliability and Fit indices for PAQ-S for total sample and sample groups (sex and country).

Sample *(n)*	Total (2535)	Spain (388)	Italy (376)	Lebanon (487)	Nigeria (561)	Türkiye (410)	Ukraine (313)	Male (678)	Female (1857)
Mean	18.99	17.39	16.86	21.84	21.90	16.08	17.66	19.27	18.88
SD	8.01	7.84	6.92	8.00	8.04	7.36	7.10	7.70	8.11
Skewness	0.47	0.65	0.71	0.37	0.11	0.61	0.61	0.37	0.50
Kurtosis	−0.30	0.60	0.68	−0.65	−0.45	−0.23	0.38	−0.37	−0.27
K-S (*z*)	3.18	1.75	1.41	1.96	0.98	2.10	1.19	1.74	2.77
*p*	<0.001	0.004	0.034	<0.001	0.331	<0.001	0.116	0.005	<0.001
F *(p)*	-	53.17 (<0.001)						1.16 (0.282)	
Correlations *								
PAQ-S/NA	0.419	0.454	0.393	0.577	0.486	0.360	0.464	-	-
PAQ-S/SI	0.406	0.310	0.334	0.621	0.269	0.394	0.368	-	-
PAQ-S/DS	0.471	0.446	0.429	0.633	0.449	0.437	0.447	-	-
Fit indices								-	-
*X^2^/df*	19.56	3.61	3.29	3.08	6.25	3.84	3.93	-	-
*p*	<0.001	<0.001	<0.001	<0.001	<0.001	<0.001	<0.001	-	-
CFI	0.97	0.98	0.97	0.99	0.95	0.97	0.95	-	-
TLI	0.96	0.97	0.95	0.98	0.92	0.95	0.92	-	-
RMSEA	0.086	0.082	0.078	0.065	0.097	0.083	0.097	-	-
SRMR	0.064	0.072	0.078	0.053	0.075	0.079	0.084	-	-
Reliability									
Coefficient α	0.83	0.87	0.83	0.85	0.76	0.82	0.79	-	-
Coefficient ω	0.83	0.87	0.82	0.84	0.77	0.81	0.79	-	-

Note: (*) The correlation is significant in all cases at the 0.01 level (two-tailed). SD = Standard deviation. NA = Negative affectivity; SI = Social Inhibition; DS = Personality Type D measured with DS14 (NA + SI). CFI = Comparative Fit Index; TLI: Tucker-Lewis Index; RMSEA = Root mean square error of approximation; SRMR = Standardized root mean square residual.

### 3.2. Reliability, fit indices and invariance analysis

The results of the descriptive statistics, correlations, reliability, and fit indices are presented in Table 1. Cronbach's α and McDonald's ω indicated good construct validity for PAQ-S (α-values > 0.80 and ω-values > 0.75) in all samples, except for the Nigerian sample, which had an α-value of 0.76. In any case, the ω-value is 0.77, which is considered acceptable.

Regarding the fit indices, the WLSMV estimator did not improve the fit indices obtained with the DWLS estimator. The data obtained suggested that the one-factor model, without correlated error terms, showed adequate fit indices for CFI/TLI but poor fit indices for RMSEA and SRMR. Correlating errors of items 3 and 6 allowed for fit adjustments in all samples (TLI and CFI > 0.90; RMSEA < 0.08; and SRMR ≤ 0.08), except in the Lebanese sample, where a TLI/CFI = 1 was obtained, so adjusting the error pairs was not recommended in this sample. For consistency and comparability across all samples, the fit indices reported in Table 1 correspond to models without correlating the error terms between items 3 and 6.

Concurrent validity, which was determined through a correlation analysis, indicated positive and adequate to good results for NA, SI, and the personality Type D in all samples. However, the Nigerian sample presented a poor result for SI factor.

Table 2 presents the results of the measurement invariance analysis across sex and country. The analysis compares four increasingly restrictive models: configural invariance (M1), metric invariance (M2), scalar invariance (M3), and strict invariance (M4). For each model, the (CFI and RMSEA are presented, along with the changes (Δ) in these indices between consecutive models (M2–M1, M3–M2, M4–M3). These changes indicate whether the scale functions equivalently across groups. According to the established criteria, a change in the CFI (ΔCFI) of ≤ 0.01 and a change in the RMSEA (ΔRMSEA) of ≤ 0.015 support measurement invariance between models.

As shown in the table, the model demonstrated an acceptable fit for both the male and female groups. Moreover, the fit indices for M1 and M2 supported configural and metric invariance, thus indicating that the underlying factor structure and factor loadings were consistent across the sexes.

Similarly, in the cross-national analysis, both a configural and metric invariance were confirmed, thus suggesting that the structure and loadings of the model were comparable across the participating countries.

On the other hand, the Turkish sample showed a strict invariance with all country's samples except from Nigeria, while the Spanish sample showed a strict invariance with samples from Lebanon, Türkiye, and Ukraine. The Italian sample showed a strict invariance with these from Türkiye and Ukraine. The Nigerian sample also showed scalar invariance from Ukraine.

**Table 2.** Measurement Invariance of the PAQ-S Across Sexes and Countries (pairs of samples).

Model comparison	CFI	ΔCFI			RMSEA	ΔRMSEA		
		M2–M1	M3–M2	M4–M3		M2–M1	M3–M2	M4–M3
Sex	0.977	0.000	**−0.015**	−0.001	0.081	−0.009	0.012	−0.007
Country								
Spain/Italy	0.979	−0.003	**−0.016**	0.002	0.080	−0.005	0.013	−0.009
Spain/Lebanon	0.986	−0.006	−0.010	−0.005	0.073	0.004	0.009	0.002
Spain/Nigeria	0.969	−0.010	**−0.013**	**−0.052**	0.091	0.005	0.001	**0.026**
Spain/Türkiye	0.977	−0.003	−0.006	−0.002	0.083	−0.004	0.000	−0.005
Spain/Ukraine	0.974	−0.005	−0.007	−0.006	0.089	−0.003	−0.000	−0.003
Italy/Lebanon	0.984	−0.005	**−0.025**	−0.004	0.071	0.001	**0.025**	−0.006
Italy/Nigeria	0.961	−0.007	**−0.014**	−0.058	0.090	−0.004	0.003	**0.024**
Italy/Türkiye	0.971	−0.007	−0.002	−0.003	0.081	−0.001	−0.005	−0.004
Italy/Ukraine	0.965	−0.010	−0.003	−0.007	0.087	0.000	−0.005	−0.003
Lebanon/Nigeria	0.975	−0.002	**−0.031**	**−0.030**	0.084	−0.007	**0.025**	0.012
Lebanon/Türkiye	0.983	−0.002	−0.011	−0.002	0.074	−0.006	0.010	−0.004
Lebanon/Ukraine	0.980	0.000	**−0.016**	−0.004	0.079	−0.009	0.015	−0.004
Nigeria/Türkiye	0.960	−0.003	**−0.012**	**−0.030**	0.091	−0.007	0.001	0.012
Nigeria/Ukraine	0.952	−0.006	−0.004	**−0.027**	0.097	−0.006	−0.006	0.009
Türkiye/Ukraine	0.963	−0.001	−0.002	0.002	0.090	−0.009	−0.006	−0.009

### 3.3. Psychometric NwA

**Figure 1.**
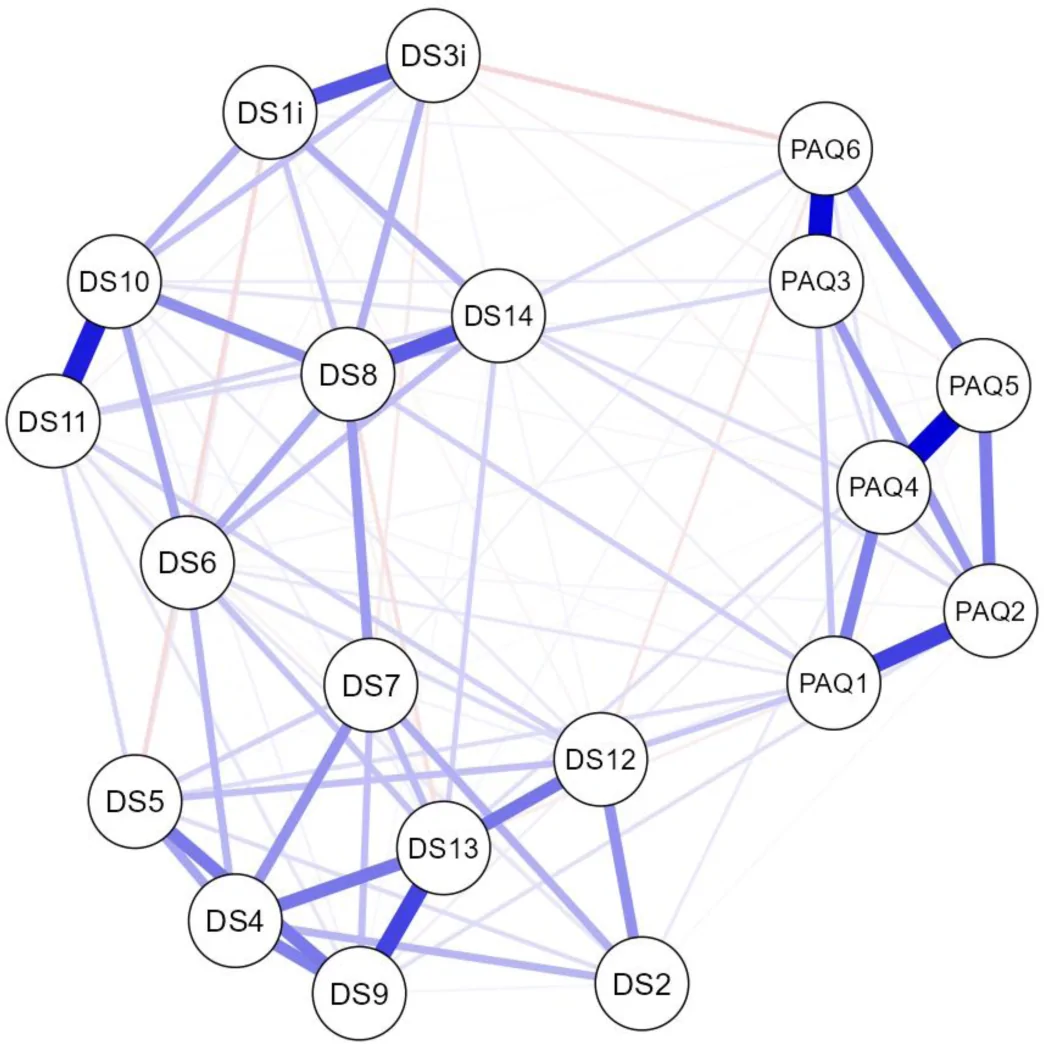
NwA for total sample. PAQ = PAQ-S items. DS = DS14 items. Blue lines indicate positive relationships and red lines indicate negative relationships, thus illustrating the associations among variables.

Figure 1 (Figure S1 in supplementary) displays the outcomes of the NwA, which included all items from both the PAQ-S and the DS14. As anticipated, the items are organized into distinct and coherent clusters, each reflecting conceptually related groupings that are aligned with the underlying constructs of the study. These clusters mirrored the theoretical latent variables, thus providing evidence that the components effectively captured their intended dimensions. Furthermore, the network structure supported the discriminant validity of the constructs, with clear differentiation between the PAQ-S items and those related to the NA and SI dimensions of a Type D personality.

The NwA stratified by sex (male and female) revealed consistent clustering patterns across both groups (Figure S2). However, the network sparsity values differed, with a sparsity of 0.479 observed in the male group and 0.384 in the female group, the latter matching the sparsity value of the total sample (Table S3). These results indicate a higher degree of interconnection between items across different scales and subscales in male compared to female. Similarly, when analyzing the NwA by country (Figure S3 to S8), a consistent structure of node groupings and cluster configurations was observed across all national samples. Nevertheless, the sparsity levels (Table S3) varied from 0.584 to 0.432, with the Lebanese sample exhibiting the densest network, followed by the Nigerian and Ukrainian samples. In contrast, the Turkish, Spanish, and Italian networks showed lower levels of item interconnectivity, thus suggesting comparatively simpler relational structures among the questionnaire items.

**Figure 2.**
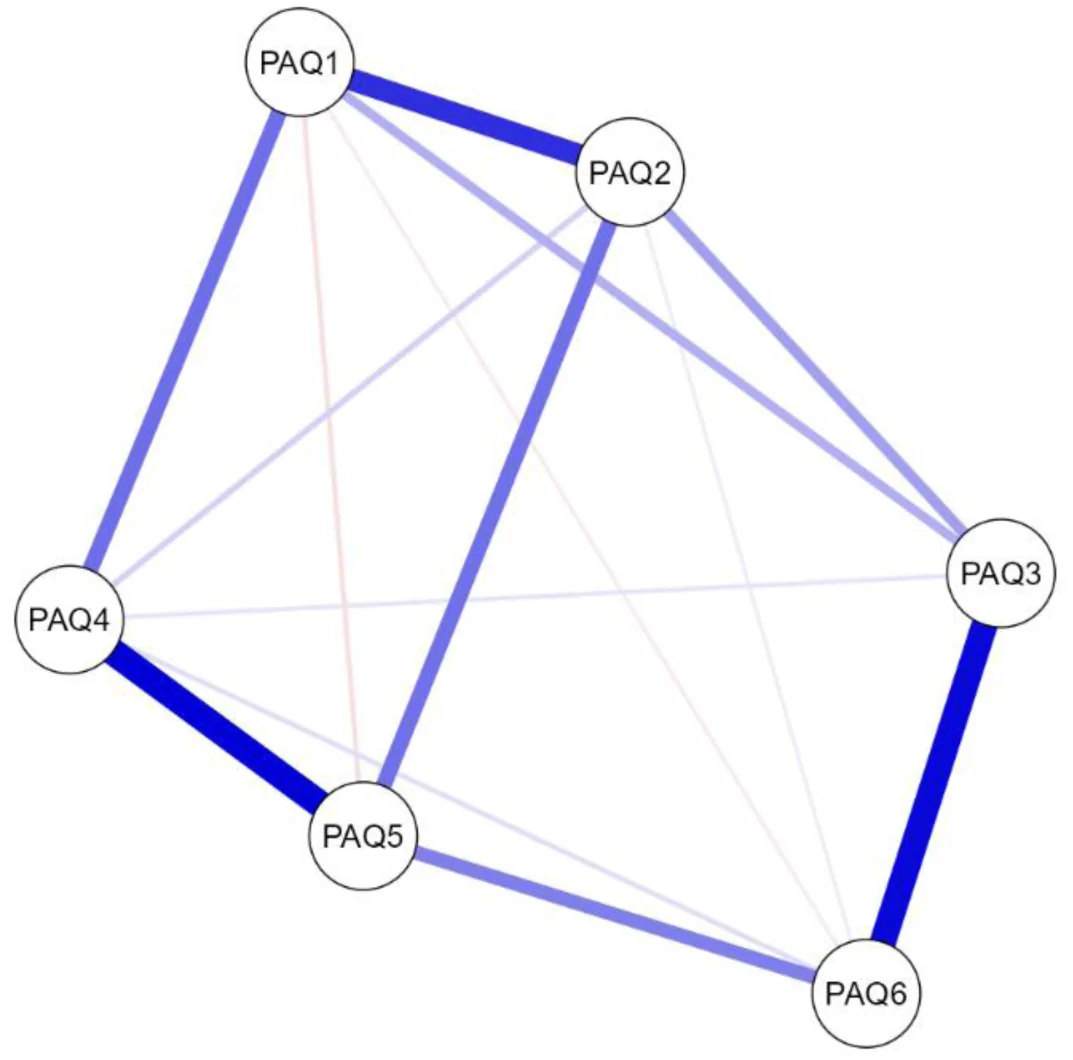
Network plot for total study sample. Note that PAQ = PAQ-S items. Blue lines indicate positive relationships, with the thickness of the lines representing the strength of the associations among variables.

**Figure 3.**
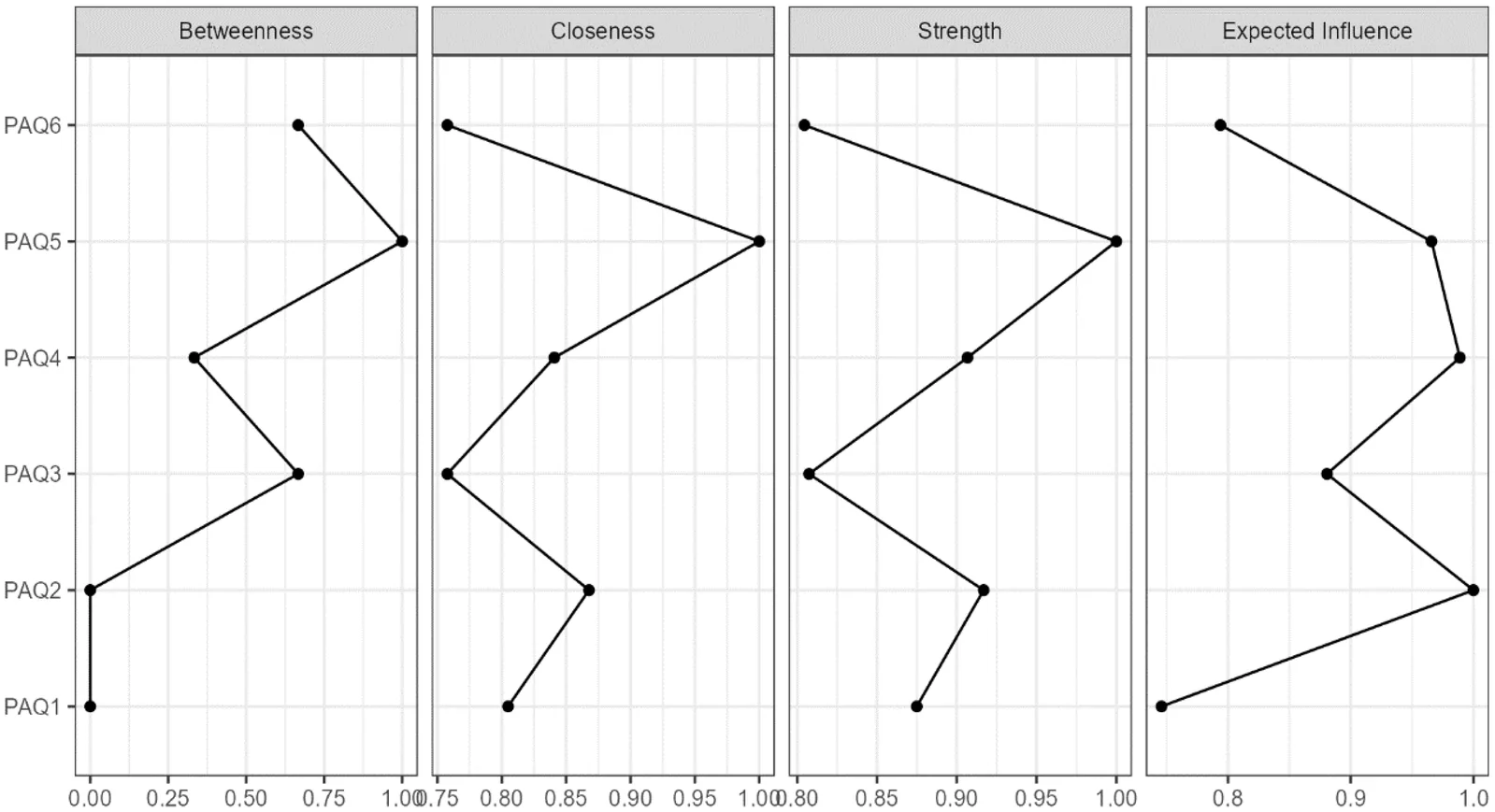
Centrality plots for total study sample that illustrate the relative importance of each item within the network using centrality measures, which help identify the most influential variables in the structure.

For the PAQ-S items, the NwA for total sample can be seen in Figure 2 (Figure S9 in supplementary). The centrality plots can be observed in Figure 3. The results of the weight matrix and centrality are presented in Table 3. The network displayed 6 nodes, with a sparsity of 0.067 (Table S4), thus indicating a clear interrelation between all analyzed items. The highest relationship weight was observed for items 1 and 2 (*r* = 0.386), 3 and 6 (*r* = 0.458), and 4 and 5 (*r* = 0.471), which is consistent with the theoretical foundations of the scale.

**Figure 4.**
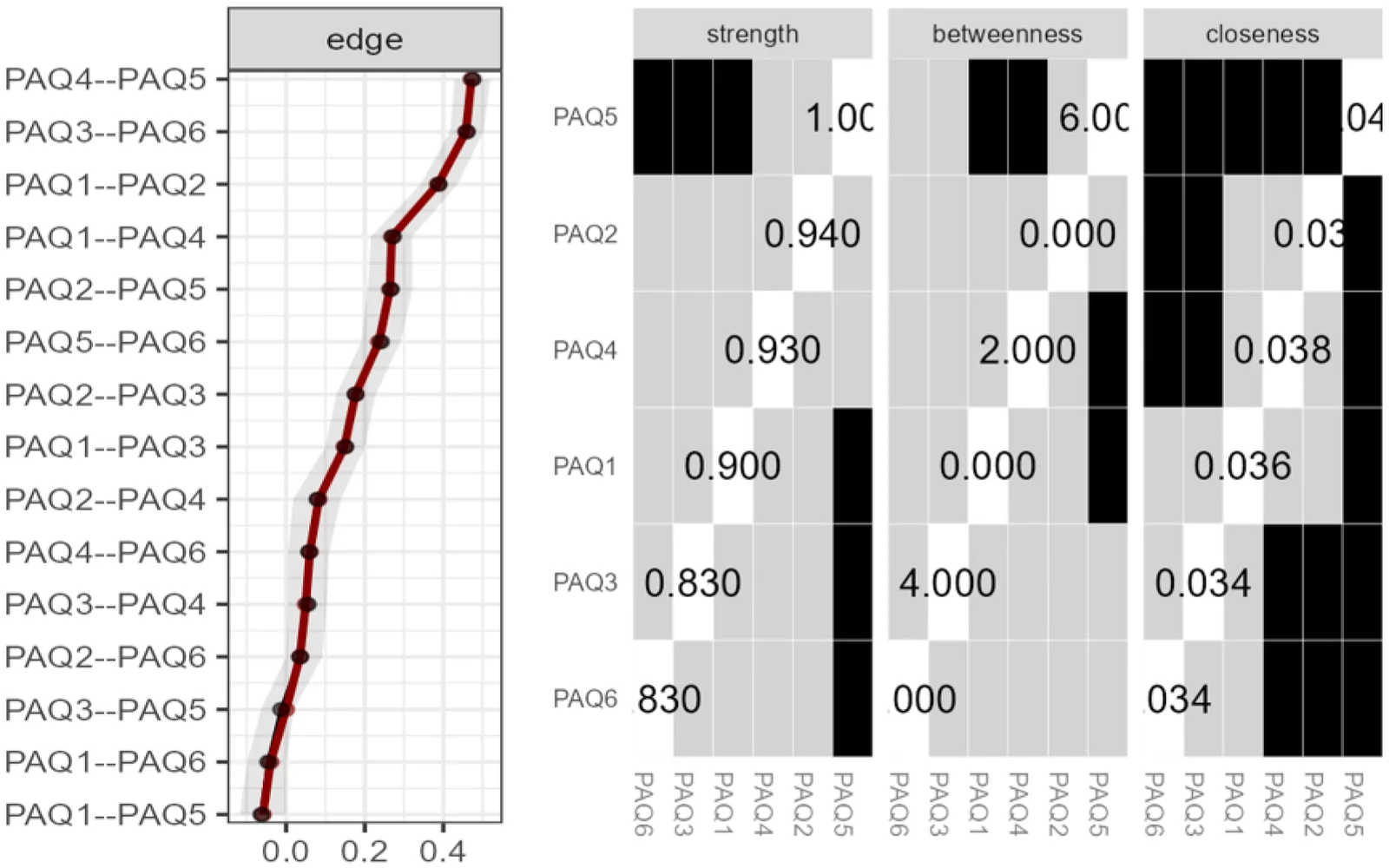
Edge stability (Left image) and Centrality stability (Right image) for total sample. In the image on the left, each horizontal line represents a network edge, with the red lines indicating the estimated edge weights and the surrounding grey bands depicting their 95% confidence intervals.

This pattern highlights the pairing of items designed to evaluate negative emotions, positive emotions, and those specifically targeting externally oriented thinking (EOT), thus underscoring the conceptual grouping intended by the scale's design. Nodes corresponding to items 2 (“When I'm feeling bad, I can't tell whether I'm sad, angry, or scared”), 4 [“When I'm feeling good (feeling a pleasant emotion), I can't find the right words to describe those feelings”], and 5 (“When I'm feeling good, I can't tell whether I'm happy, excited, or amused”) showed the highest expected influence, with the node corresponding to item 5 having the highest centrality. Items 3 (“I tend to ignore how I feel”) and 6 (“I don't pay attention to my emotions”) were nodes with an adequate betweenness index. The stability analyses are shown in Figure 4. The edge stability was adequate in all cases. The stability of the strength centrality indices was good for all nodes. Betweenness showed good stability for nodes corresponding to items 3 and 5, but not for the node corresponding to item 6.

The results, for the PAQ-S items, for every gender (as male/female) and country can be seen separately in the supplementary. Table S4 presents the sparsity data, Table S5 shows the weight matrix, and Table S6 provide the centrality indices. The network plots for each group (sex and country) are presented in Figures S10 and S11, and centrality plots in Figures S12 to S14. Figures S15 to S22 included the edge stability and the centrality stability for all group.

**Table 3.** Edge weights and node centrality metrics in the total sample, which shows the strength of connections between items (edge weights) and the relative importance of each item within the network based on centrality measures.

Variable	**Weight matrix**						**Centrality measures**			
	PAQ1	PAQ2	PAQ3	PAQ4	PAQ5	PAQ6	Betweenness	Closeness	Strength	Expected influence
PAQ1	0.000						−1.101	−0.367	−0.137	−1.399
PAQ2	**0.386**	0.000					−1.101	0.327	0.430	**0.971**
PAQ3	0.148	0.176	0.000				**0.550**	−0.887	−1.051	−0.141
PAQ4	0.269	0.083	0.050	0.000			−0.275	0.030	0.293	**0.868**
PAQ5	−0.059	0.264	0.000	**0.471**	0.000		**1.376**	**1.784**	**1.555**	**0.652**
PAQ6	−0.040	0.035	**0.458**	0.061	0.236	0.000	**0.550**	−0.887	−1.090	−0.951

In the NwA, depending on the culture, all samples, except the Lebanese sample, presented a weight matrix between items 1 (“When I'm feeling bad (feeling an unpleasant emotion), I can't find the right words to describe those feelings”) and 2, 3 and 6, and 4 and 5, as expected. However, Spain and Türkiye showed low correlation values between item 1 and item 2, as their load was divided with item 3 and item 4. The Lebanese sample showed the expected correlations, but the items had complex correlations with most items, thus distributing their load and presenting low weights in the matrix (<0.400 in all cases). Additionally, in all samples, except the Italian sample, item 5 showed good values for all four centrality indices. The Lebanese and Turkish samples also presented good centrality indices for item 4, with item 4 having a greater expected influence than item 5. Additionally, the Nigerian and Ukrainian samples showed good centrality indices for item 2, with item 2 having a greater expected influence than item 5 in the Nigerian sample, but not in the Ukrainian sample, where the indices reached no significant values (<0.500). The Italian sample only showed good centrality indices for item 2. The stability resulted in edges and the centrality confirmed the validity of the findings for these samples.

Similarly, the gender-based analysis revealed an identical structural pattern, although there was a greater sparsity observed in the male sample (0.267) compared to the female sample (0.133), thus suggesting a greater interrelation between the items of the scale in females than in males. Similarly, the highest relationship weight was observed for items 1 and 2 (*r* = 0.292/0.407), 3 and 6 (*r* = 0.353/0.484), and 4 and 5 (*r* = 0.441/477). The nodes corresponding to items 2, 4, and 5 showed the highest expected influence, and the node corresponding to item 5 has the highest centrality.

## 4. Discussion

The CFA indicated that the PAQ-S exhibited a satisfactory univariate structure in the analyzed samples, which supports the first hypothesis.

Although the adequate fit indices were obtained without the need to correlate error pairs, these notably improved in all samples (except the Lebanese one) when correlating the error terms of items 3 and 6, thus aligning with previous findings where it was also necessary to correlate this pair of items [13],[32],[33]. However, in those studies, it was also necessary to correlate the error pairs of items 1 and 2 and items 4 and 5 to achieve an adequate model fit. These differences could be attributed to cultural nuances in the interpretation of the items, as well as sample-specific characteristics. Therefore, although our results generally align with previous research, they reflect some particularities unique to our data.

The NwA revealed a clear differentiation between the PAQ-S and DS14 items, thus indicating that the construct of alexithymia measured by PAQ-S was distinct from the Type D personality traits. This finding aligns with earlier studies (e.g.: [23]–[25]) which suggested independence between these constructs and confirms the second hypothesis of this study.

The PAQ-S showed a good internal consistency (Cronbach's α > 0.80, McDonald's ω > 0.75) in all samples except the Nigerian sample, where the α was marginally lower (0.76). However, the remaining parameters were adequate, thus indicating that the instrument had an overall acceptable reliability. These results confirm the third hypothesis and are consistent with the reliability indices reported in previous research [13],[32],[33].

Significant positive correlations were observed between the alexithymia scores and the Type D personality markers, thus supporting our fourth hypothesis. This finding is in line with prior research studies (e.g.: [15],[17]) and indicates good correlations between these constructs.

In line with Larionow et al. [32], by applying CTT, the PAQ-S demonstrated metric invariance across genders, thus indicating that the scale functions similarly for male and female. Metric invariance was also observed between cultures. Türkiye, Spain, and Italy showed a strict invariance with certain countries, but not with others. Consequently, the fifth hypothesis has only been partially confirmed in this way.

By applying the NwA, it was observed that, in general, the countries showed correlation matrices as expected for the PAQ-S, thus indicating that the questionnaire items are consistently measuring the construct of alexithymia. A similar structure is also observed regarding the items that exhibit centrality, with notable consistency in the centrality of item 5, which appears as a central node in most networks, followed by either item 2 or item 4. Thus, although most countries show similar patterns of correlation and centrality, some specific differences stand out in individual items and their relative centrality. As indicated by Chan et al. [28] and Munroe and Munroe [31], these differences may reflect cultural variations in how the PAQ-S items are interpreted and responded to. According to Dion [26], Konrath et al. [29], and Ng & Chan [30], Asian countries report higher levels of alexithymia compared to Western countries.

In our study, Lebanon and Nigeria reported higher levels than the other countries. However, the Turkish report values that are similar to European countries. A key characteristic of Lebanese Arab culture is that it is collectivist and values social harmony [56]. However, it is a self-assertive culture, which is similar to Western cultures [57]. Therefore, while they value social cohesion, they also value expressing their emotions to some extent [58]. Another characteristic of Lebanese culture is its dualistic perception of control [59]. These dualistic beliefs about control may be partly due to religion, which leads to the belief that, on the one hand, individuals can control their own outcomes, while on the other hand, outcomes are beyond their control [59]. Thus, although emotions are felt intensely [58], they may not be openly expressed [56], which could create an environment in which alexithymia is more prevalent. Nigeria could also be described as an interdependent and collectivist culture, where self-control, emotional moderation, and an adherence to social norms are highly valued [31], which may explain the presence of high levels of alexithymia. On the other hand, the results of our study align with those of Boukar and Dane [60], who detected a high prevalence rate of alexithymia among the Nigerian university students. On the other hand, Türkiye showed values similar to those of European countries, which is particularly interesting. The Turkish society exhibits both collectivist and individualist tendencies, depending on the context. In general, it tends to be collectivist, especially in rural settings and in family and community relationships. However, in urban centers and certain aspects of life, such as work and education, it shows individualist influences [61]. Therefore, the obtained results could be explained by the sample used in the study.

While the study provided valuable insights into cultural differences in alexithymia, it is essential to recognize that the measurement of alexithymia may vary across cultural contexts. Different populations may have distinct thresholds for reporting emotional difficulties, and the tools used to measure alexithymia might not be equally valid across all cultures. Moreover, socioeconomic factors, educational levels, and the access to psychological support systems may also affect how alexithymia is expressed or reported. Therefore, future research should explore these aspects in greater depth to clarify the underlying mechanisms.

The findings of this study have important practical implications for both public health and clinical settings across diverse cultural contexts. The demonstrated reliability and validity of the PAQ-S in multiple countries suggest that it can serve as a robust tool to screen alexithymia in varied populations. Clinicians can use the PAQ-S to identify individuals who may have difficulties with emotional awareness and expression, which is crucial to effectively tailor psychological interventions. Furthermore, understanding cultural differences in alexithymia levels and item interpretation can guide the development of culturally sensitive assessment protocols and treatment plans. In public health, the early detection of alexithymia using the PAQ-S may facilitate targeted mental health promotion and prevention strategies, particularly in populations at higher risks due to cultural or socioeconomic factors. Overall, the PAQ-S represents a valuable instrument to enhance the emotional health assessment within global mental health initiatives.

### 4.1. Study limitations

The first limitation of this study concerns the sample, which consists of university students and is predominantly female. Studies with other samples (e.g., the general population, clinical samples) and across different cultures are needed. The sample composition bias—where 73.3% of participants were female—should be considered. Although measurement invariance was tested by gender, the implications of this distribution were not explicitly addressed in the recruitment process. This bias may have influenced sex-related findings, and therefore, the results should be interpreted with caution in this regard. Second, it is a cross-sectional study that does not allow one to establish construct causality. Third, the test-retest reliability of the PAQ-S was not examined, nor were comparisons made with other alexithymia scales. Lastly, another limitation was the use of a self-report measure administered online, which might have resulted in biases related to poor self-awareness or social desirability. Another important limitation of this study is that, due to the voluntary participation and broad, non-stratified dissemination, it was not possible to control or ensure a balanced representation of students from different fields of study in each country. This means that specific data on the distribution by academic discipline are not available, which could affect the generalizability of the results. Future research could consider a more stratified sampling approach to explore potential differences across academic areas. Therefore, it is recommended to continue researching this topic with different samples and cultures to optimize the administration of measurement tools adapted to each situation.

## 5. Conclusions

The PAQ-S has shown strong psychometric properties in the different cultures analyzed, as well as between sexes, thus achieving the main objectives of the study and providing a solid basis for future cross-cultural research regarding alexithymia. The supplementary provided additional details on the stability and centrality indices, thus reinforcing the validity of our findings.

## Use of AI tools declaration

The authors declare they have not used Artificial Intelligence (AI) tools in the creation of this article.


