# Supplementary material for: Psychometric properties and measurement invariance of the Perth Alexithymia Questionnaire Short Form (PAQ-S) in Arabic, English, Italian, Spanish, Turkish, and Ukrainian
Source: AIMS Public Health. 2025 Aug 13;12(3):835–56. doi: 10.3934/publichealth.2025042 (PMC12538251; doi:10.3934/publichealth.2025042)
Supplement: Supplementary file 1 [file publichealth-12-03-042-s001.pdf]

Public Health

AIMS Public Health, 12(3): 835–856.
DOI: 10.3934/publichealth.2025042
Received: 07 May 2025
Revised: 01 July 2025
Accepted: 16 July 2025
Published: 13 August 2025

https://www.aimspress.com/journal/aimsph

Research article

Psychometric properties and measurement invariance of the Perth Alexithymia Questionnaire Short Form (PAQ-S) in Arabic, English, Italian, Spanish, Turkish, and Ukrainian

Olga Malas[1,*], Giulia Colombini[2], Anastasiia Shyroka[3], Dayo Omotoso[4], Asiye-Şengül Avşar[5], Nada Mallah Boustani[6], Mirko Duradoni[2] and Angel Blanch[1]

[1] Department of Psychology, Sociology and Social Work, University of Lleida, Avinguda de l'Estudi General, 4, 25001 Lleida, Spain
[2] Department of Education, Languages, Interculture, Literatures and Psychology, University of Florence, Via di San Salvi, 12, Building 26, 50135 Florence, Italy
[3] Department of Psychology and Psychotherapy, Ukrainian Catholic University, Sventsitskogo 17, 79011 Lviv, Ukraine
[4] Department of Human Anatomy, Redeemer's University, Ede, Osun State, Nigeria
[5] Department of Measurement and Evaluation in Education, Recep Tayyip Erdoğan University, Campus Zihni Derin - Fener Mahallesi 53100 Rize, Türkiye
[6] Faculty of business and management, Saint Joseph University, PO BOX 17-5208 Mar Mikhael, 1104 2020 Beirut, Lebanon

* **Correspondence:** Email: olga.malas@udl.cat.

Table S1. The PAQ-S Questionnaire.

Item	Sentence
1	When I'm feeling bad (feeling an unpleasantemotion), I can't find the right words todescribe those feelings.
2	When I'm feeling bad, I can't tell whetherI'm sad, angry, or scared.
3	I tend to ignore how I feel.
4	When I'm feeling good (feeling a pleasantemotion), I can't find the right words todescribe those feelings.
5	When I'm feeling good, I can't tell whetherI'm happy, excited, or amused.
6	I don't pay attention to my emotions.

Table S2. Descriptive statistics for PAQ-S items.

**PAQ1**	**Spain**	**Italy**	**Lebanon**	**Nigeria**	**Türkiye**	**Ukraine**
Mean	3.518	3.428	3.756	4.380	3.244	3.492
Std. Deviation	1.729	1.681	1.793	1.964	1.620	1.589
**PAQ2**						
Mean	2.631	2.763	3.493	3.886	2.651	2.958
Std. Deviation	1.616	1.566	1.820	2.091	1.664	1.695
**PAQ3**						
Mean	3.131	2.814	3.694	3.936	2.698	3.220
Std. Deviation	1.748	1.637	1.674	1.942	1.805	1.734
**PAQ4**						
Mean	2.871	3.109	3.651	3.499	2.727	3.000
Std. Deviation	1.746	1.635	1.763	2.035	1.735	1.774
**PAQ5**						
Mean	2.418	2.524	3.678	3.187	2.410	2.553
Std. Deviation	1.582	1.495	1.815	1.969	1.673	1.709
**PAQ6**						
Mean	2.820	2.223	3.569	3.016	2.349	2.438
Std. Deviation	1.613	1.445	1.726	1.916	1.639	1.602

Network Analysis for PAQ-S ítems & DS14 items

Table S3. Summary of Network for PAQ-S & DS14.

	Number of nodes	Number of non-zero edges	Sparsity
Total sample	20	117/190	0.384
Male	20	99/190	0.479
Female	20	117/190	0.384
Country			
1	20	82 / 190	0.568
2	20	79 / 190	0.584
3	20	108 / 190	0.432
4	20	94 / 190	0.505
5	20	84 / 190	0.558
6	20	95 / 190	0.500

Note: 1= Spain. 2= Italy. 3= Lebanon. 4= Nigeria. 5= Türkiye. 6 =Ukraine. PAQ: Alexithymia measured with PAQ-S. DS: Personality Type D measured with DS14.

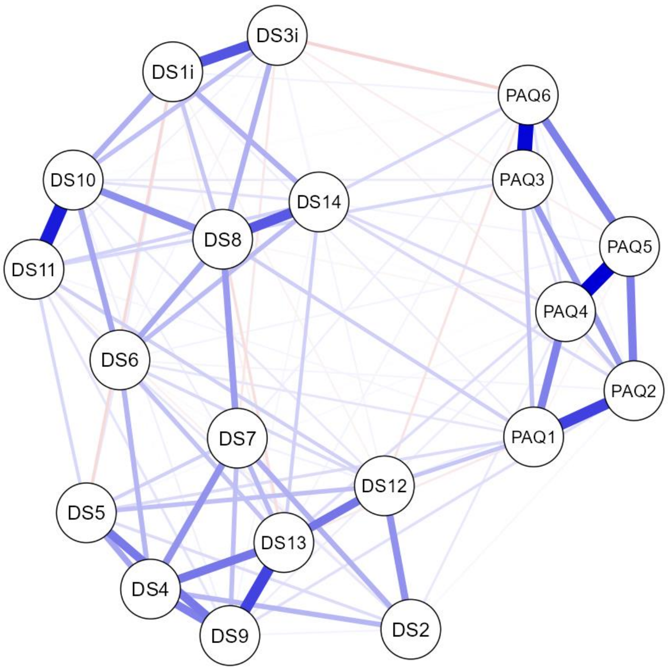

Figure S1. Network plot for total sample. *Note: PAQ: Alexithymia measured with PAQ-S. DS: Personality Type D measured with DS14.*

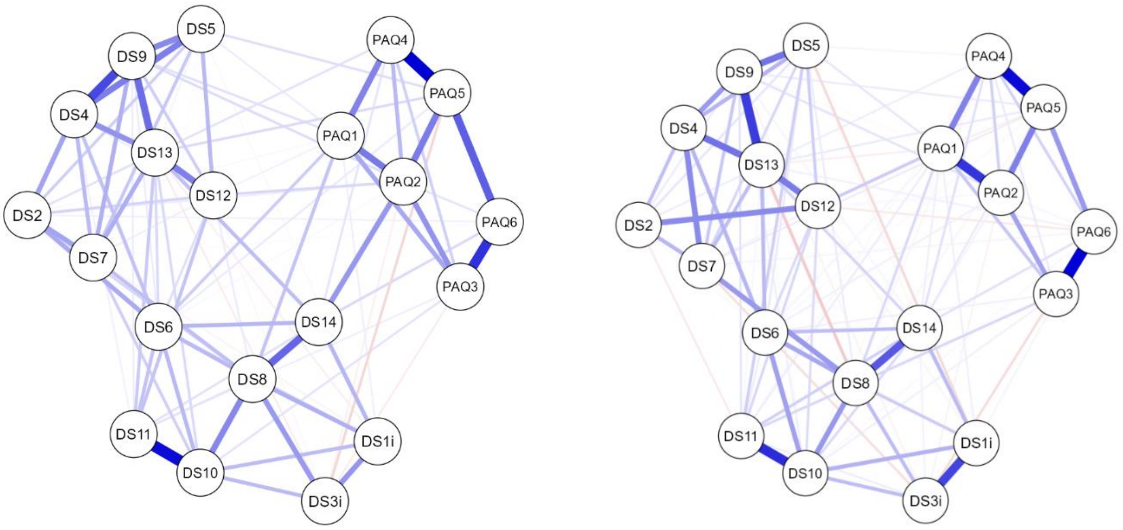

Figure S2. Male & Female network plot. *Note: PAQ: Alexithymia measured with PAQ-S. DS: Personality Type D measured with DS14.*

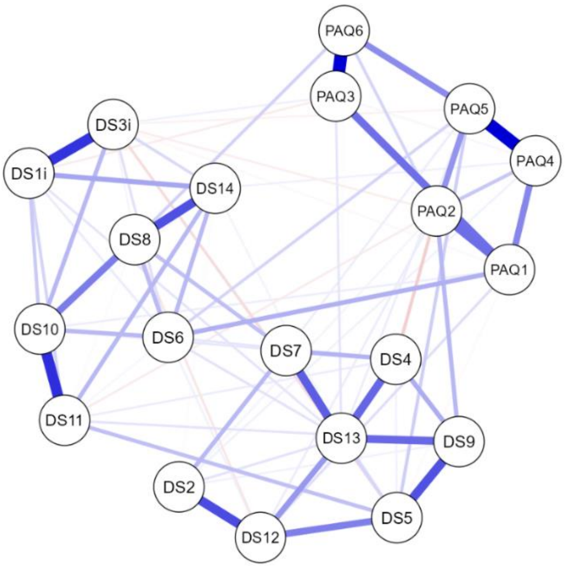

Figure S3. Network plot for spanish sample. Note: PAQ: Alexithymia measured with PAQ-S. DS: Personality Type D measured with DS14.

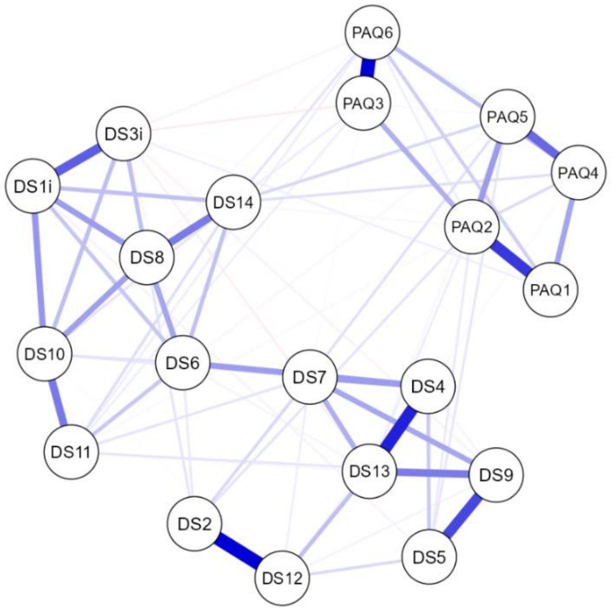

Figure S4. Network plot for italian sample. *Note: PAQ: Alexithymia measured with PAQ-S. DS: Personality Type D measured with DS14.*

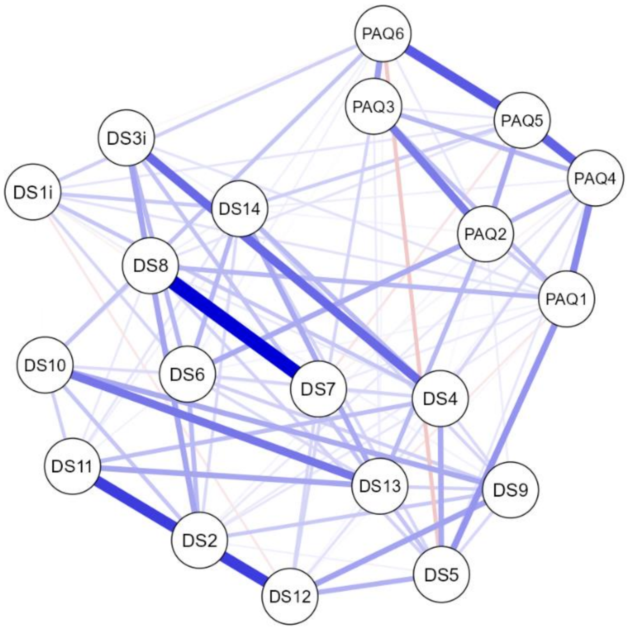

Figure S5. Network plot for lebanese sample. *Note that: PAQ: Alexithymia measured with PAQ-S. DS: Personality Type D measured with DS14.*

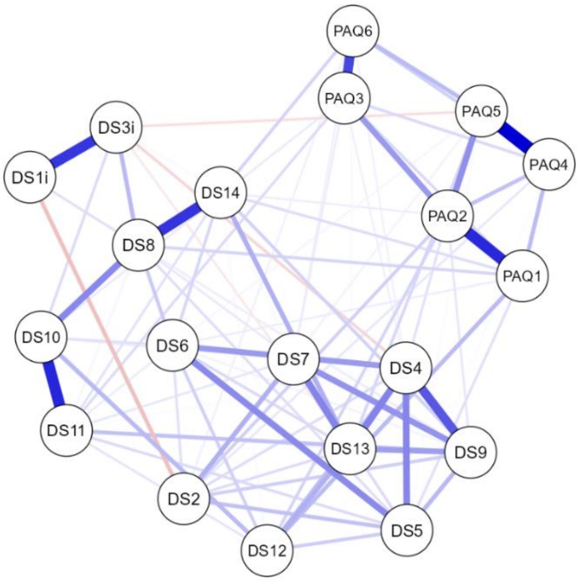

Figure S6. Network plot for nigerian sample. *Note: PAQ: Alexithymia measured with PAQ-S. DS: Personality Type D measured with DS14.*

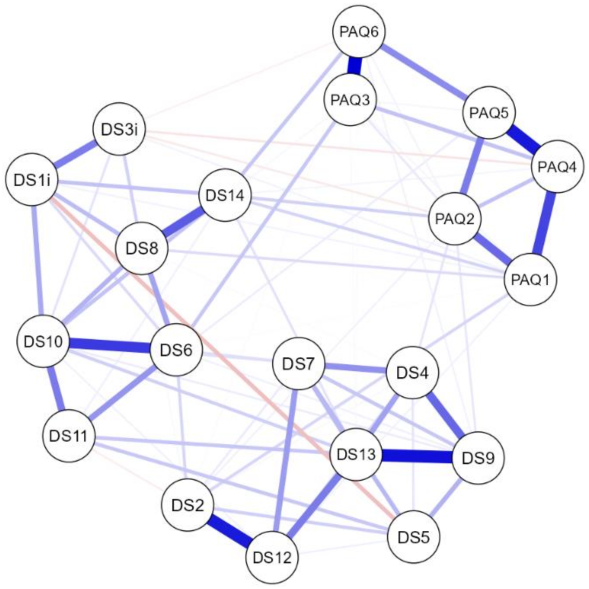

Figure S7. Network plot for turkish sample. *Note that: PAQ: Alexithymia measured with PAQ-S. DS: Personality Type D measured with DS14.*

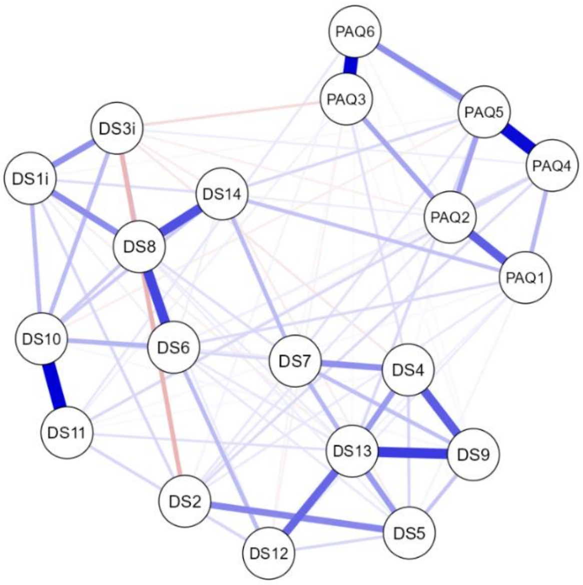

Figure S8. Network plot for ukrainian sample. *Note: PAQ: Alexithymia measured with PAQ-S. DS: Personality Type D measured with DS14.*

Network Analysis for PAQ-S items

Table S4. Summary of Network for PAQ-S.

Group	Nodes	Non-zero Edges	Sparsity
General	6	14/15	0.067
Male	6	11/15	0.267
Female	6	13/15	0.133
Spain	6	11/15	0.267
Italy	6	12/15	0.200
Lebanon	6	14/15	0.067
Nigeria	6	15/15	0.000
Türkiye	6	14/15	0.067
Ukraine	6	12/15	0.200

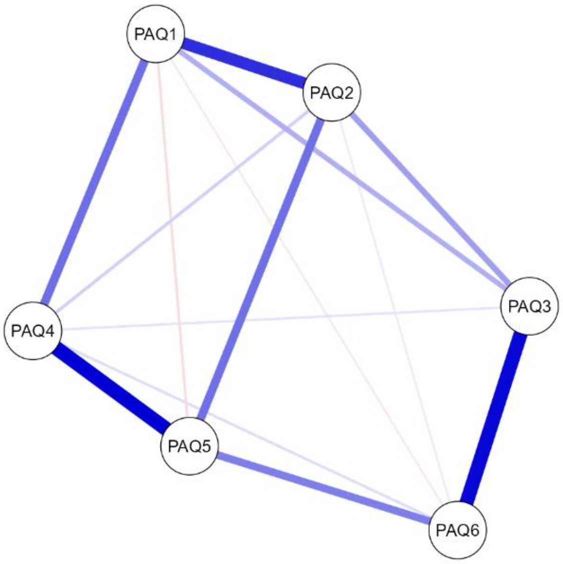

Figure S9. Network plot for total sample.

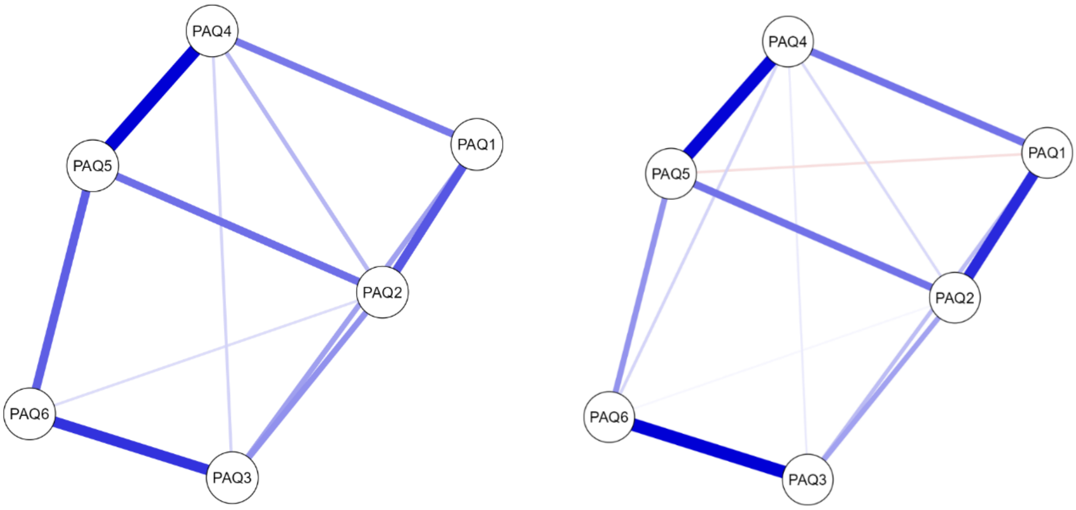

Figure S10. Network plot for male and female sample.

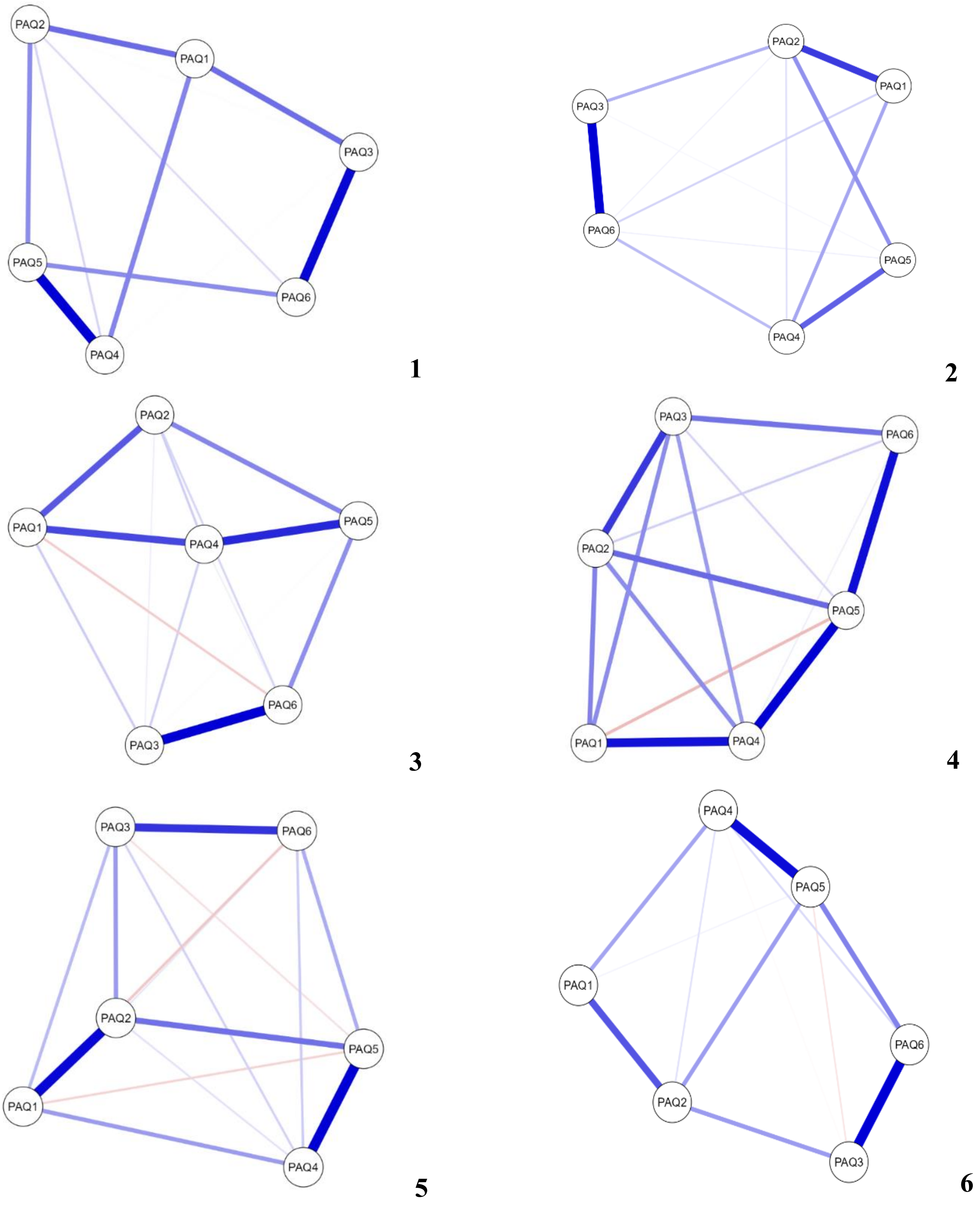

Figure S11. Network plots. *Note: 1= Spain. 2= Italy. 3= Lebanon. 4= Nigeria. 5= Türkiye. 6 =Ukraine.*

Table S5. Weights matrix per group and variable.

Group	Variable	PAQ1	PAQ2	PAQ3	PAQ4	PAQ5	PAQ6
Total	PAQ1	0.000					
	PAQ2	**0.386**	0.000				
	PAQ3	0.148	0.176	0.000			
	PAQ4	0.269	0.083	0.050	0.000		
	PAQ5	−0.059	0.264	0.000	**0.471**	0.000	
	PAQ6	−0.040	0.035	**0.458**	0.061	0.236	0.000
Male	PAQ1	0.000					
	PAQ2	0.292	0.000				
	PAQ3	0.165	0.189	0.000			
	PAQ4	0.233	0.125	0.074	0.000		
	PAQ5	0.000	0.250	0.000	0.441	0.000	
	PAQ6	0.000	0.061	**0.353**	0.000	0.279	0.000
Female	PAQ1	0.000					
	PAQ2	**0.407**	0.000				
	PAQ3	0.128	0.178	0.000			
	PAQ4	0.268	0.072	0.040	0.000		
	PAQ5	−0.062	0.269	0.000	**0.477**	0.000	
	PAQ6	0.000	0.015	**0.484**	0.082	0.205	0.000
Spain	PAQ1	0.000					
	PAQ2	0.320	0.000				
	PAQ3	0.315	0.005	0.000			
	PAQ4	0.266	0.091	0.004	0.000		
	PAQ5	0.000	0.249	0.000	**0.552**	0.000	
	PAQ6	0.000	0.076	**0.535**	0.000	0.250	0.000
Italy	PAQ1	0.000					
	PAQ2	**0.471**	0.000				
	PAQ3	0.000	0.189	0.000			
	PAQ4	0.211	0.077	0.000	0.000		
	PAQ5	0.000	0.262	0.016	0.380	0.000	
	PAQ6	0.105	0.031	**0.608**	0.162	0.035	0.000
Lebanon	PAQ1	0.000					
	PAQ2	0.181	0.000				
	PAQ3	0.166	**0.287**	0.000			
	PAQ4	**0.344**	0.164	0.143	0.000		
	PAQ5	−0.100	0.216	0.061	0.368	0.000	
	PAQ6	0.000	0.068	0.202	0.025	0.349	0.000
Nigeria	PAQ1	0.000					

Group	Variable	PAQ1	PAQ2	PAQ3	PAQ4	PAQ5	PAQ6
	PAQ2	**0.476**	0.000				
	PAQ3	0.132	0.194	0.000			
	PAQ4	0.191	0.056	0.084	0.000		
	PAQ5	−0.082	0.275	−0.059	**0.489**	0.000	
	PAQ6	−0.105	0.047	0.389	0.098	0.159	0.000
Türkiye	PAQ1	0.000					
	PAQ2	0.350	0.000				
	PAQ3	0.105	0.034	0.000			
	PAQ4	**0.386**	0.099	0.097	0.000		
	PAQ5	0.000	0.255	0.004	0.454	0.000	
	PAQ6	−0.103	0.084	**0.535**	0.031	0.235	0.000
Ukraine	PAQ1	0.000					
	PAQ2	0.370	0.000				
	PAQ3	0.000	**0.218**	0.000			
	PAQ4	0.198	0.062	−0.007	0.000		
	PAQ5	0.025	0.214	−0.054	0.532	0.000	
	PAQ6	0.000	0.000	**0.555**	0.078	0.271	0.000

Table S6. Centrality measures per group and variable.

Group	Variable	Betweenness	Closeness	Strength	Expected Influence
Total	PAQ1	−1.101	−0.367	−0.137	−1.399
	PAQ2	−1.101	0.327	0.430	0.971
	PAQ3	0.550	−0.887	−1.051	−0.141
	PAQ4	−0.275	0.030	0.293	0.868
	PAQ5	**1.376**	**1.784**	**1.555**	**0.652**
	PAQ6	0.550	−0.887	−1.090	−0.951
Men	PAQ1	−0.886	−0.722	−1.112	−1.112
	PAQ2	−0.886	0.217	0.825	0.825
	PAQ3	−0.221	−0.732	−0.333	−0.333
	PAQ4	−0.221	−0.088	0.440	0.440
	PAQ5	**1.771**	**1.889**	**1.266**	**1.266**
	PAQ6	0.443	−0.564	−1.085	−1.085
Women	PAQ1	−1.101	−0.514	−0.375	−1.382
	PAQ2	−1.101	**0.811**	**0.540**	**1.048**
	PAQ3	0.550	−0.938	−0.787	−0.295
	PAQ4	−0.275	0.057	**0.522**	**1.030**
	PAQ5	**1.376**	**1.522**	**1.404**	0.417
	PAQ6	0.550	−0.938	−1.305	−0.819
Spain	PAQ1	0.645	**1.330**	0.132	0.132

Group	Variable	Betweenness	Closeness	Strength	Expected Influence
	PAQ2	−1.291	−1.345	−1.454	−1.454
	PAQ3	−0.323	−0.264	−0.287	−0.287
	PAQ4	−0.323	−0.258	0.252	0.252
	PAQ5	**1.614**	1.025	**1.628**	**1.628**
	PAQ6	−0.323	−0.488	−0.271	−0.271
Italy	PAQ1	−1.088	−0.059	−0.516	−0.516
	PAQ2	**1.814**	**1.757**	**1.517**	**1.517**
	PAQ3	0.363	−0.677	−0.299	−0.299
	PAQ4	−0.363	0.431	−0.159	−0.159
	PAQ5	−0.363	−0.399	−1.313	−1.313
	PAQ6	−0.363	−1.053	0.770	0.770
Lebanon	PAQ1	−0.791	−0.616	−0.609	−1.351
	PAQ2	−0.791	−0.138	0.149	0.527
	PAQ3	−0.791	−0.845	−0.198	0.195
	PAQ4	0.791	1.085	0.927	**1.271**
	PAQ5	**1.581**	**1.392**	**1.220**	0.400
	PAQ6	0.000	−0.879	−1.490	−1.042
Nigeria	PAQ1	−0.761	−0.022	0.382	−0.950
	PAQ2	**1.523**	**1.528**	0.968	**1.503**
	PAQ3	−0.190	−0.938	−0.817	−0.233
	PAQ4	−0.761	−0.492	−0.256	0.768
	PAQ5	0.952	0.840	**1.116**	0.005
	PAQ6	−0.761	−0.915	−1.392	−1.093
Türkiye	PAQ1	−0.429	−0.195	0.183	−0.929
	PAQ2	−0.945	−0.469	−0.947	−0.262
	PAQ3	−0.945	−1.226	−1.379	−0.629
	PAQ4	0.086	0.601	**1.330**	**1.672**
	PAQ5	**1.632**	**1.656**	0.222	0.731
	PAQ6	0.601	−0.367	0.590	−0.584
Ukraine	PAQ1	−1.614	−1.565	−1.663	−1.592
	PAQ2	0.323	0.482	0.019	0.304
	PAQ3	0.323	−0.157	−0.172	−0.759
	PAQ4	−0.645	−0.107	0.092	0.293
	PAQ5	**1.291**	**1.503**	**1.458**	**1.172**
	PAQ6	0.323	−0.157	0.265	0.581

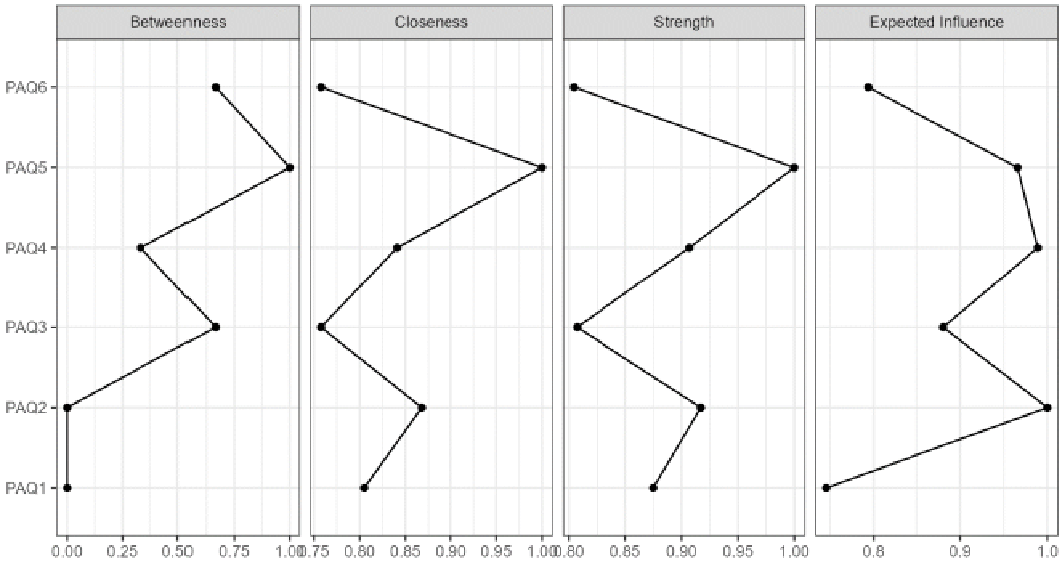

Figure S12. Centrality Plot for toral sample.

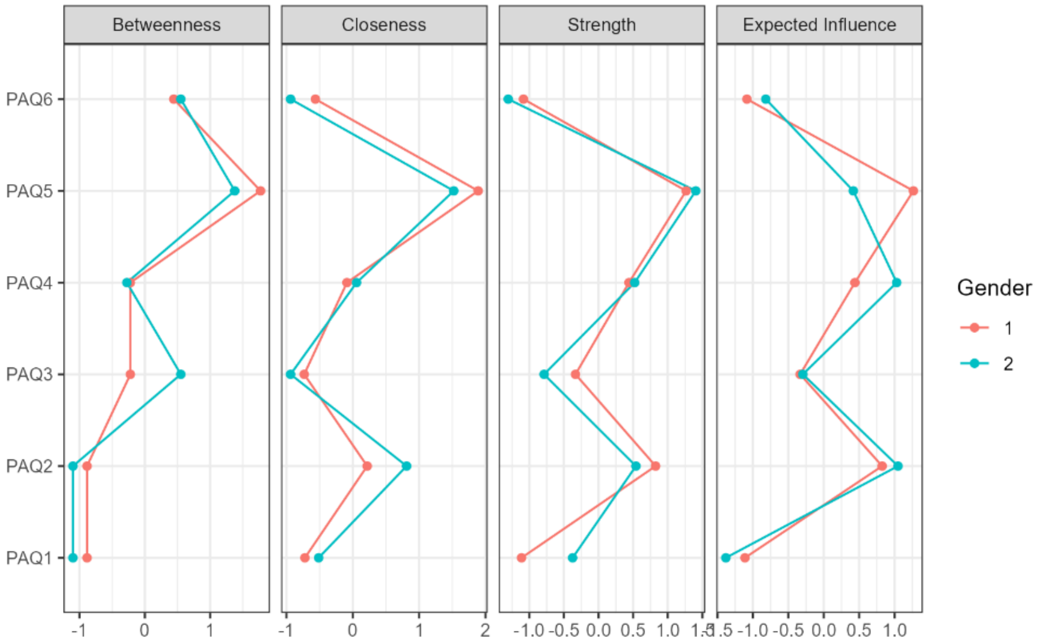

Figure S13. Centrality Plot for male (1) and female (2) samples.

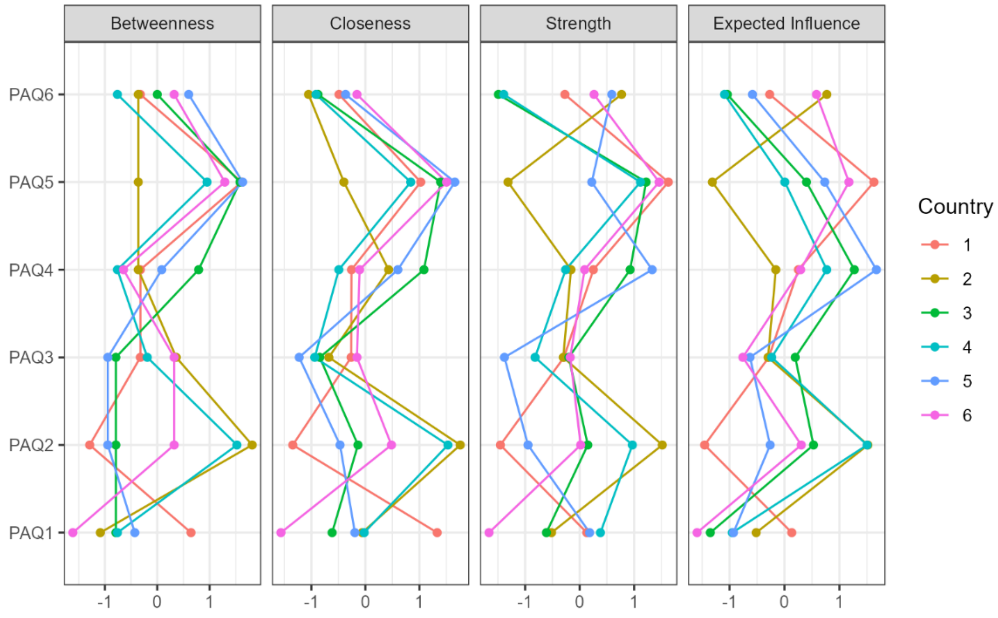

Figure S14. Centrality Plot by country. *Note: 1= Spain. 2= Italy. 3= Lebanon. 4= Nigeria. 5= Türkiye. 6 =Ukraine.*

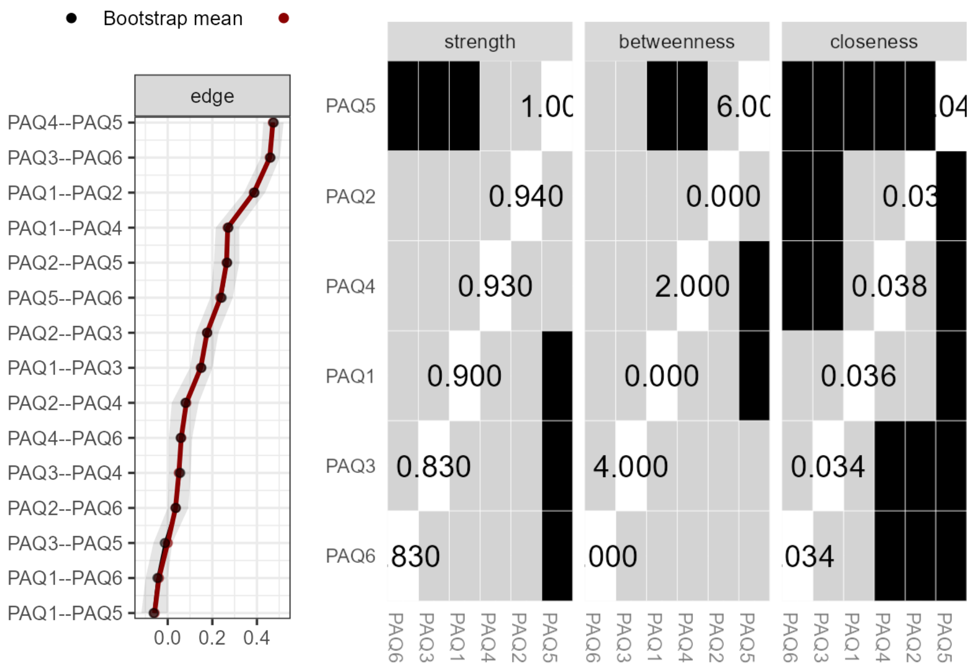

Figure S15. Edge stability (first image) and centrality stability (second image) for total sample. [It is suggested that the CS coefficient should not be below 0.25 and preferably should be above 0.5. According Epskamp et al. (2018) a correlation stability coefficient of 0.7 or higher is a useful threshold].

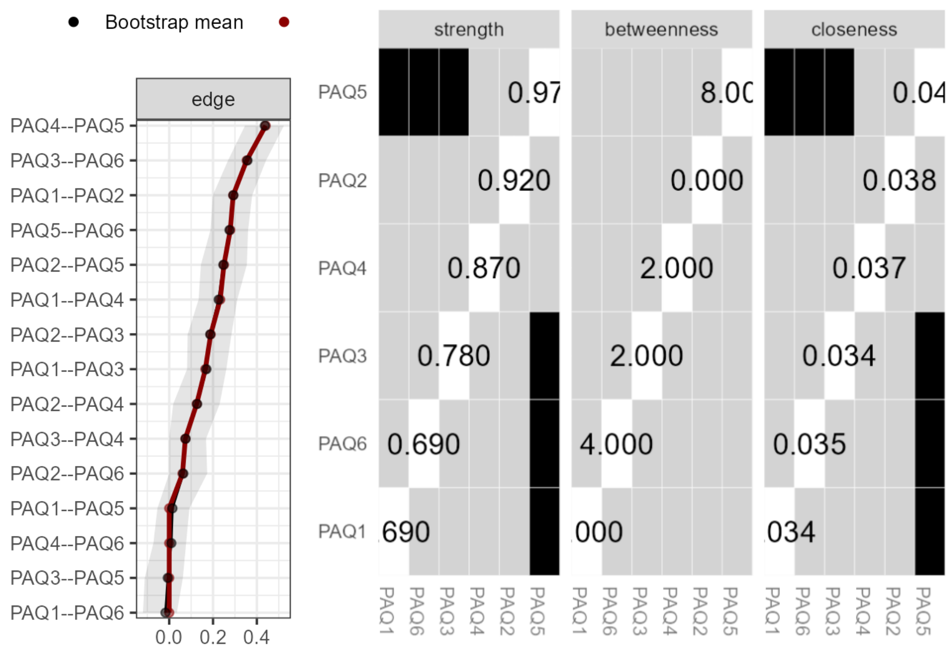

Figure S16. Edge stability (first image) and centrality stability (second image) for male sample. [It is suggested that the CS coefficient should not be below 0.25 and preferably should be above 0.5. According Epskamp et al. (2018) a correlation stability coefficient of 0.7 or higher is a useful threshold].

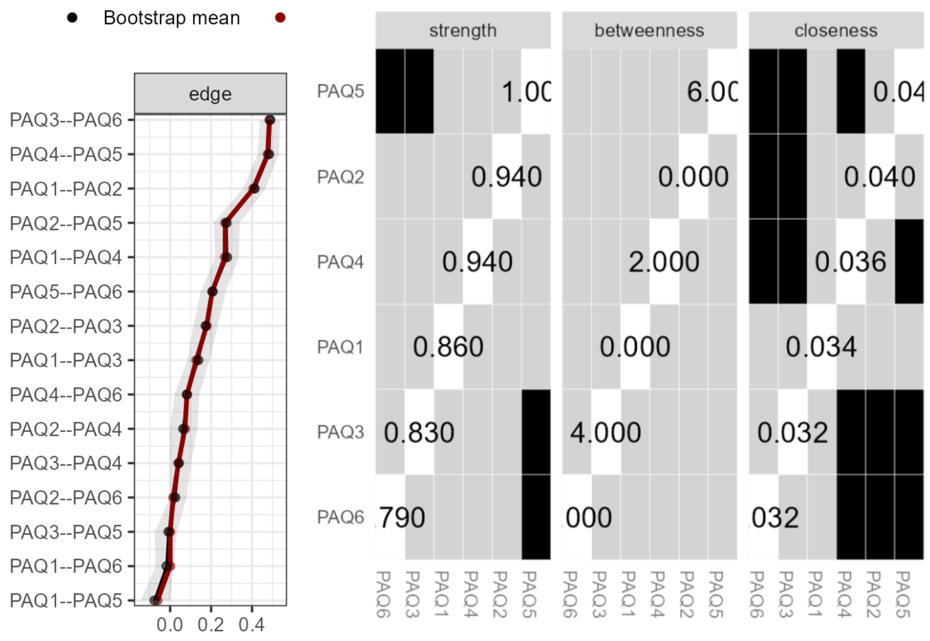

Figure S17. Edge stability (first image) and centrality stability (second image) for female sample. [It is suggested that the CS coefficient should not be below 0.25 and preferably should be above 0.5. According Epskamp et al. (2018) a correlation stability coefficient of 0.7 or higher is a useful threshold].

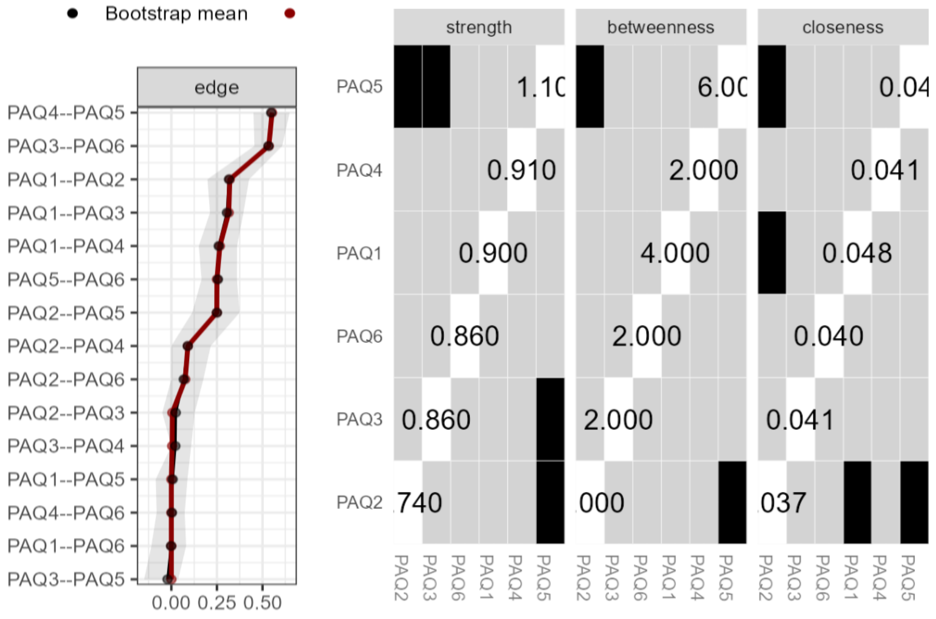

Figure S18. Edge stability (first image) and centrality stability (second image) for Spanish sample. [It is suggested that the CS coefficient should not be below 0.25 and preferably should be above 0.5. According Epskamp et al. (2018) a correlation stability coefficient of 0.7 or higher is a useful threshold].

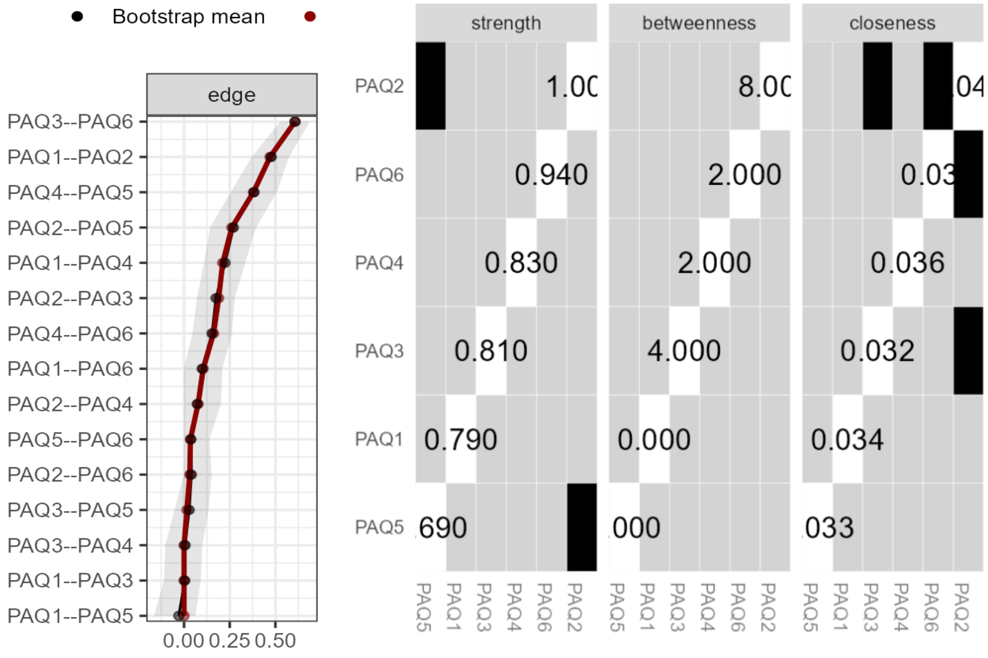

Figure S19. Edge stability (first image) and centrality stability (second image) for Italian sample. [It is suggested that the CS coefficient should not be below 0.25 and preferably should be above 0.5. According Epskamp et al. (2018) a correlation stability coefficient of 0.7 or higher is a useful threshold].

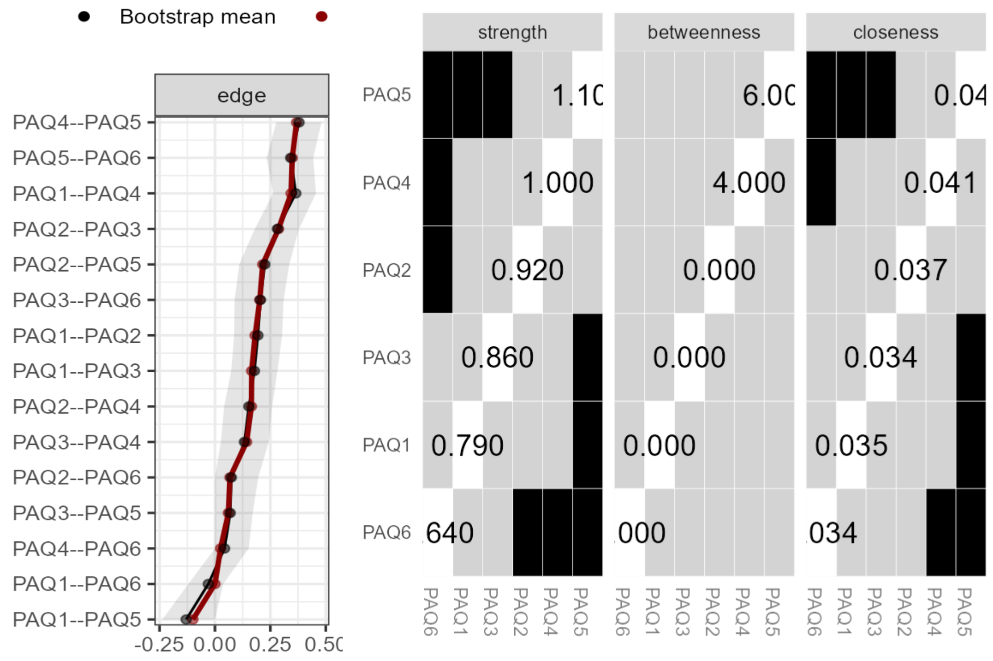

Figure S20. Edge stability (first image) and centrality stability (second image) for Lebanese sample. [It is suggested that the CS coefficient should not be below 0.25 and preferably should be above 0.5. According Epskamp et al. (2018) a correlation stability coefficient of 0.7 or higher is a useful threshold].

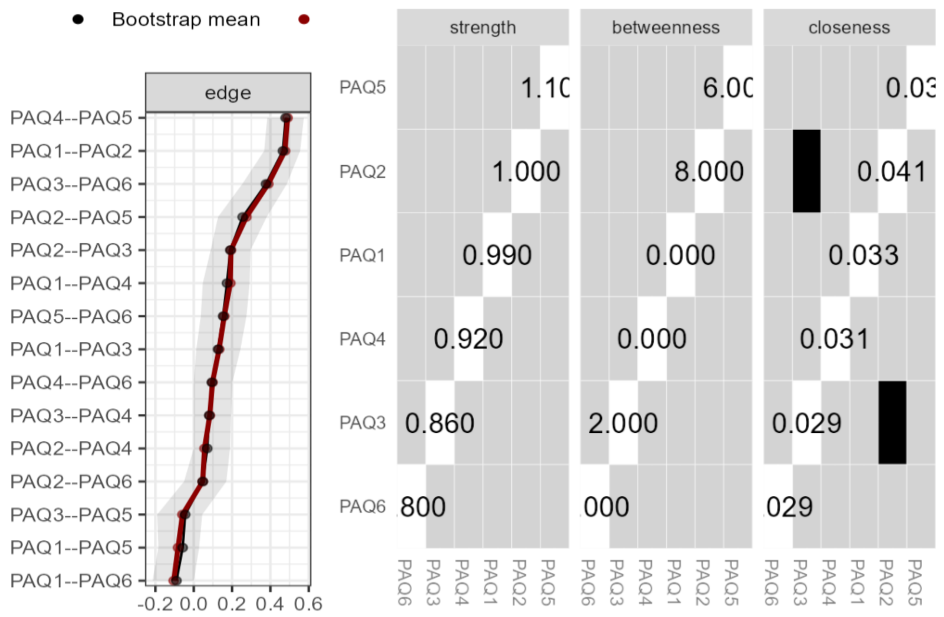

Figure S21. Edge stability (first image) and centrality stability (second image) for Nigerian sample. [It is suggested that the CS coefficient should not be below 0.25 and preferably should be above 0.5. According Epskamp et al. (2018) a correlation stability coefficient of 0.7 or higher is a useful threshold**].**

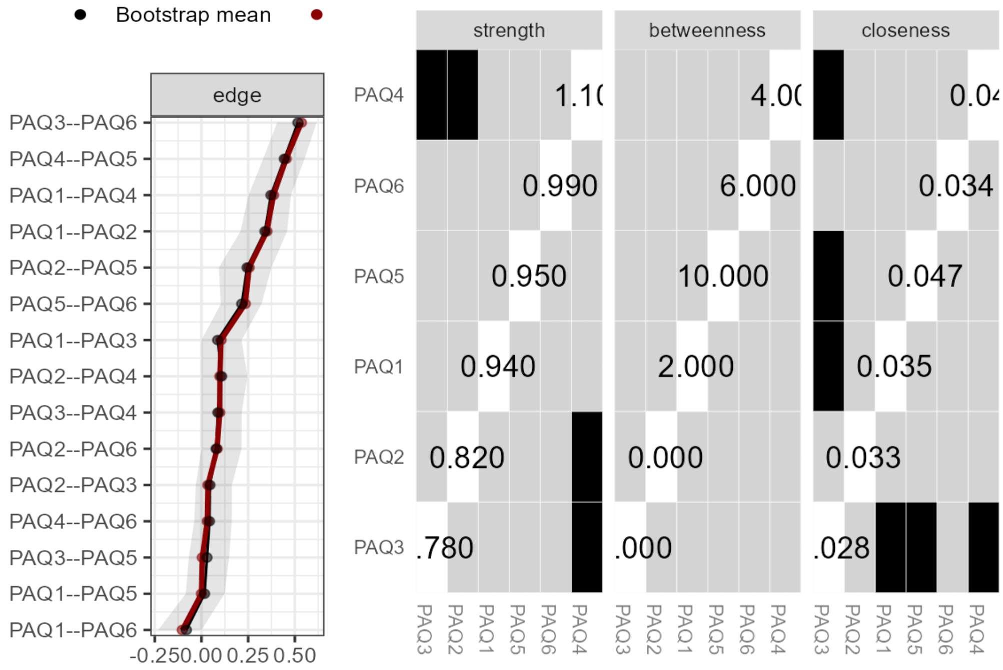

Figure S22. Edge stability (first image) and centrality stability (second image) for Turkish sample. [It is suggested that the CS coefficient should not be below 0.25 and preferably should be above 0.5. According Epskamp et al. (2018) a correlation stability coefficient of 0.7 or higher is a useful threshold].

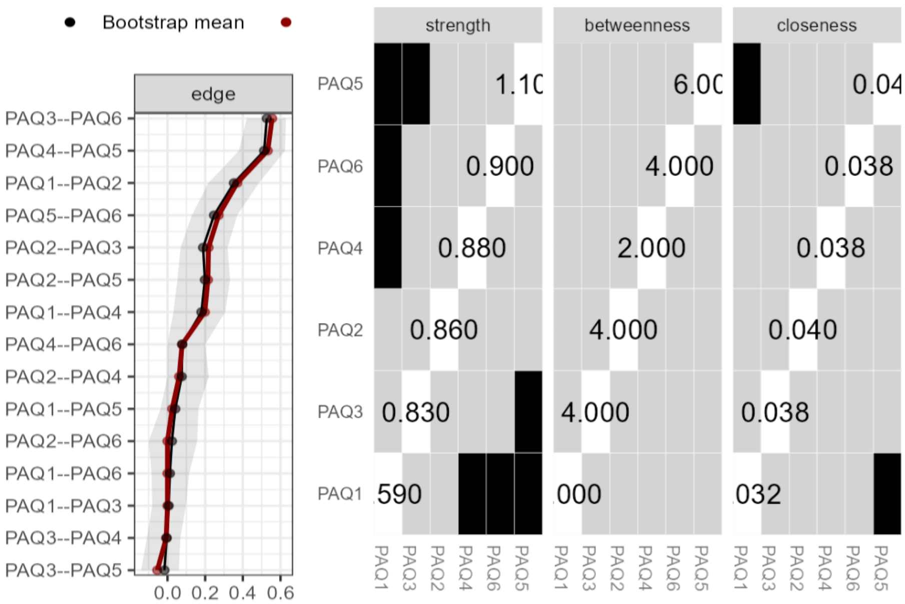

Figure S23. Edge stability (first image) and centrality stability (second image) for Ukrainian sample. [It is suggested that the CS coefficient should not be below 0.25 and preferably should be above 0.5. According Epskamp et al. (2018) a correlation stability coefficient of 0.7 or higher is a useful threshold].

AIMS Press

© 2025 the Author(s), licensee AIMS Press. This is an open access article distributed under the terms of the Creative Commons Attribution License (https://creativecommons.org/licenses/by/4.0)
